# Loss of Intestinal MSTN Promotes GLP‐1 Secretion Through SGLT1 Palmitoylation

**DOI:** 10.1002/advs.78302

**Published:** 2026-10-14

**Authors:** Sheng‐Zhong Han, Shuang‐Yan Chang, Zhaobo Luo, Ke Sun, Biaohu Quan, Yanqiu Lv, Zhouyan Li, Xinyue Liu, Sitong Lv, Meifu Xuan, Xiaoyufan Xu, Xue Fang, Hang Chang, Rihuan Zhang, Xi‐Jun Yin, Lin‐Hu Quan, Jin‐Dan Kang

**Affiliations:** ^1^ Department of Animal Science Yanbian University Yanji Jilin People's Republic of China; ^2^ Key Laboratory of Natural Medicines of the Changbai Mountain College of Pharmacy Ministry of Education Yanbian University Yanji People's Republic of China; ^3^ Department of Medicine Beth Israel Deaconess Medical Center and Harvard Medical School Boston Massachusetts USA; ^4^ Jilin Provincial Key Laboratory of Transgenic Animal and Embryo Engineering Yanbian University Yanji Jilin People's Republic of China; ^5^ Department of Emergency Guangzhou Institute of Emergency Medicine the Second Affiliated Hospital Guangzhou Medical University Guangzhou People's Republic of China

**Keywords:** GLP‐1, glucose homeostasis, myostatin, SGLT1, S‐palmitoylation

## Abstract

Intestinal glucagon‐like peptide‐1 (GLP‐1) is secreted by intestinal L cells in a glucose‐dependent manner. The localization and surface abundance of glucose‐sensing proteins on the cell membrane affect the response to glucose, but the regulatory mechanism remains poorly understood. Here, we show that S‐palmitoylation is required for stable plasma membrane enrichment of SGLT1 and for SGLT1‐mediated GLP‐1 secretion. Notably, myostatin (MSTN) was identified as a key upstream regulator of this modification. Intestinal MSTN deficiency markedly enhanced SGLT1 palmitoylation, increased its plasma membrane abundance, and thereby promoted glucose‐stimulated GLP‐1 secretion. Importantly, simvastatin induced intestinal MSTN upregulation and impaired glucose homeostasis, whereas MSTN deficiency largely alleviated these effects. Mechanistically, MSTN regulated the expression of the palmitoyl acyltransferase ZDHHC20, which catalyzed SGLT1 S‐palmitoylation at Cys301. Blocking SGLT1 palmitoylation attenuated SGLT1 membrane enrichment and markedly suppressed the induction of GLP‐1 secretion caused by MSTN deficiency. Collectively, our research results have revealed a new mechanism by which MSTN regulates intestinal endocrine function and S‐palmitoylation modification promotes GLP‐1 secretion, providing potential targets for regulating endogenous GLP‐1 secretion in the intestine and treating metabolic diseases related to glucose imbalance.

## Graphical abstract

Intestinal MSTN deficiency enhances glucose‐stimulated GLP‐1 secretion and glucose tolerance in high‐fat diet‐fed mice. Mechanistically, loss of MSTN promotes YY1‐associated ZDHHC20 expression, increasing SGLT1 S‐palmitoylation at Cys301 and its plasma membrane localization in L cells. Additionally, simvastatin increases intestinal MSTN expression, impairing GLP‐1 secretion and glucose homeostasis.

## 1 Introduction

Myostatin (MSTN) is not only a well‐established regulator of skeletal muscle development, but is also closely associated with metabolic processes including obesity, insulin resistance, and impaired glucose homeostasis [1, 2]. Our previous study showed that MSTN mutation markedly alters the intestinal microenvironment [3], suggesting that MSTN may exert previously unrecognized functions in the intestine. Statins are widely used for the primary and secondary prevention of cardiovascular disease [4]. Despite their well‐established cardiovascular benefits, increasing attention has been paid to statin‐associated new‐onset diabetes and impaired glucose tolerance [5, 6]. Previous studies have shown that statins can affect MSTN expression, although these studies have focused primarily on skeletal muscle [7]. However, whether MSTN directly participates in the regulation of intestinal metabolic and endocrine functions, and whether such effects contribute to pharmacologically induced glucose metabolic stress, remains unclear.

Intestinal L cells contribute to the regulation of postprandial glucose homeostasis by sensing nutrient signals and releasing glucagon‐like peptide‐1 (GLP‐1) [8]. The successful application of GLP‐1 receptor agonists in the treatment of type 2 diabetes and obesity, together with the association between enhanced endogenous GLP‐1 secretion and metabolic improvement after bariatric surgery, further highlights the physiological and therapeutic significance of endogenous GLP‐1 regulation [9, 10]. Nevertheless, upstream regulatory mechanisms controlling glucose sensing and endogenous GLP‐1 secretion in intestinal L cells remain incompletely understood. The involvement of sodium‐glucose cotransporter 1 (SGLT1) in glucose‐stimulated GLP‐1 secretion has been well established [11, 12], whereas the mechanisms that regulate the functional state of SGLT1 remain poorly understood. As a membrane‐localized glucose transporter, SGLT1 must maintain sufficient cell‐surface abundance to effectively engage extracellular glucose and support glucose‐sensing function [13]. Therefore, the functional availability of SGLT1 at the plasma membrane is an important determinant of glucose sensing in L cells.

Post‐translational modifications are important molecular mechanisms that regulate the localization and functional state of membrane proteins [14]. Among them, S‐palmitoylation is a dynamic and reversible lipid modification in which palmitate is attached to cysteine residues, thereby altering protein hydrophobicity and membrane affinity and regulating subcellular localization, cell‐surface abundance, and protein function [15, 16]. This modification is mainly catalyzed by members of the ZDHHC family of protein acyltransferases and is broadly involved in the localization, trafficking, and functional regulation of membrane proteins [17]. Glucose sensing in intestinal L cells depends on membrane‐localized sensing and transport proteins, and regulation of membrane localization and surface abundance may therefore influence glucose‐responsive GLP‐1 secretion [18].

Whether the cell‐surface abundance of SGLT1 is regulated by S‐palmitoylation remains unknown. In the present study, we systematically evaluated the role of MSTN in intestinal endocrine regulation and investigated the post‐translational mechanisms controlling SGLT1 membrane availability. We found that ZDHHC20 catalyzes S‐palmitoylation of SGLT1 at Cys301, promoting its plasma membrane enrichment and enhancing glucose responsiveness in L cells. Moreover, MSTN deficiency enhanced this modification through upregulation of ZDHHC20 expression, thereby promoting GLP‐1 secretion.

## 2 Results

### 2.1 MSTN Deficiency Induces GLP‐1 Secretion in HFD‐Fed Mice

We found that MSTN was detectable in the small intestine, with prominent localization in epithelial cells along the villus axis (Figure 1A,B). Interestingly, Intestinal MSTN expression was further elevated by HFD feeding relative to chow controls (Figure 1A–C; Figure S1A). To investigate whether intestinal MSTN contributes to HFD‐associated glucose metabolic dysfunction, we generated global MSTN knockout (*Mstn^−/−^*) mice and confirmed efficient deletion of MSTN (Figure 1D–F; Figure S1B–D). After 12 weeks of HFD feeding, *Mstn^−/−^* mice exhibited an expanded population of intestinal GLP‐1^+^ cells compared with wild‐type (WT) littermates (Figure 1G). Consistent with this histological finding, *Mstn^−/−^* mice displayed a stronger circulating active GLP‐1 response after glucose stimulation than WT mice (Figure 1H). Meanwhile, the expression of GLP‐1 biosynthesis‐related genes *Gcg* and *Pcsk1* was upregulated, whereas *Gip* and *Dpp4* expression remained unchanged (Figure 1I). Consistently, intestinal proglucagon/GCG‐immunoreactive protein and PC1/3 protein levels were also increased in *Mstn^−/−^* mice (Figure S1D). In line with the increased GLP‐1 response, *Mstn^−/−^* mice displayed enhanced serum insulin levels following glucose stimulation (Figure 1J). By contrast, circulating GIP, another major incretin hormone, was not significantly altered (Figure S1E), indicating that MSTN deficiency preferentially potentiates the GLP‐1‐associated incretin response.

**FIGURE 1:**
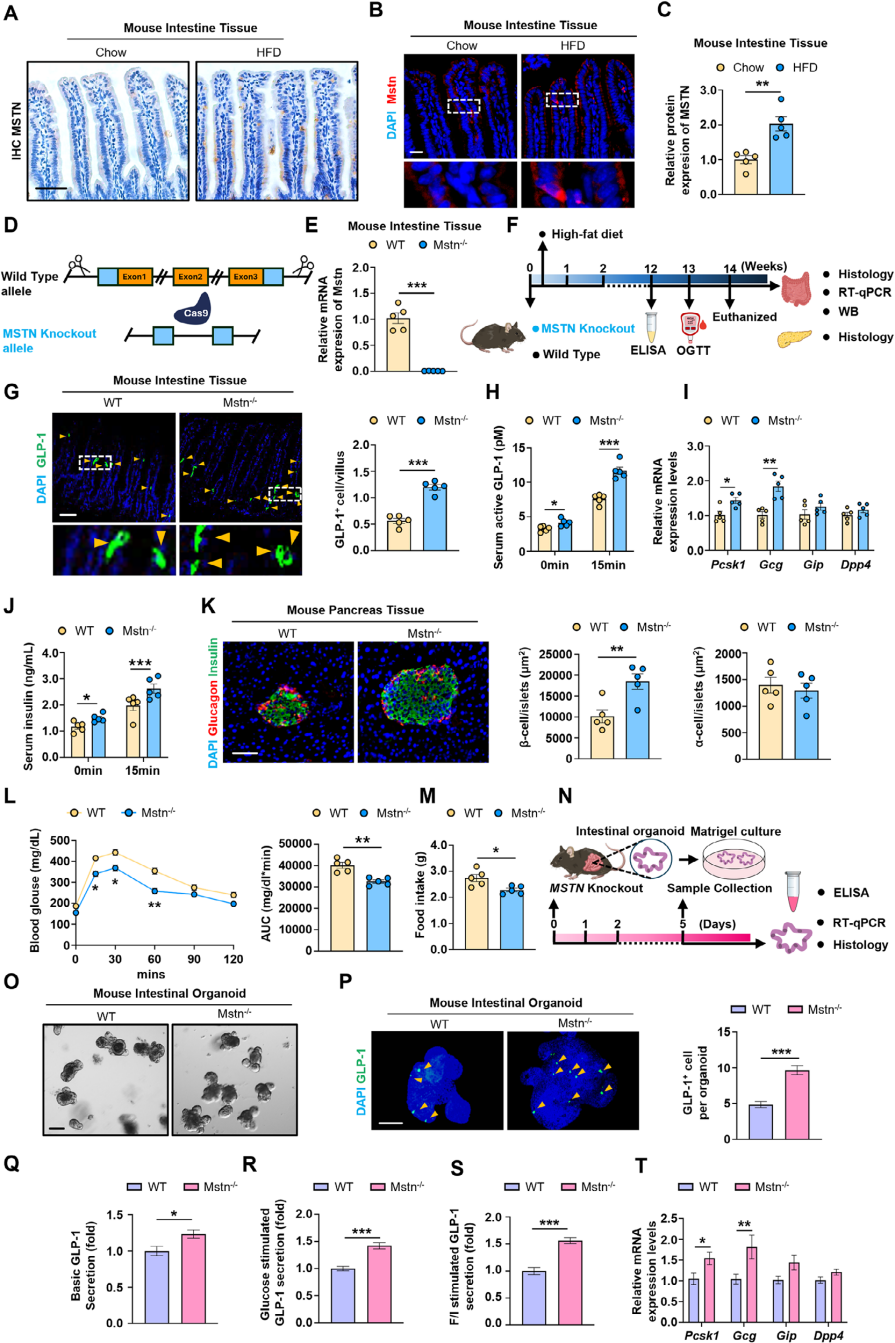
Loss of MSTN promotes HFD‐induced intestinal GLP‐1 secretion in mice. (A–C) Immunohistochemistry (A, Scale bar, 50 µm), Fluorescence in situ hybridization (FISH) (B, Scale bar, 20 µm), and immunoblot analysis (C) of MSTN expression in the small intestine of chow diet‐fed and HFD‐fed mice (*n* = 5, **p < 0.01). (D) Schematic diagram illustrating the generation of global MSTN knockout mice. (E) *Mstn* expression in the small intestine of WT and *Mstn^−/−^* mice (*n* = 5, ***p < 0.001). (F) Schematic diagram of the experimental design for HFD feeding in WT and *Mstn^−/−^* mice. (G) Representative images of GLP‐1 immunofluorescence staining in small intestine sections. Scale bar, 50 µm (*n* = 5, ***p < 0.001). (H) Serum active GLP‐1 levels before (0 min) and 15 min after oral glucose administration (*n* = 5, *p < 0.05, ***p < 0.001). (I) Relative mRNA expression of *Pcsk1*, *Gcg*, *Gip*, and *Dpp4* in the small intestine of WT and *Mstn^−/−^* mice (*n* = 5, *p < 0.05, **p < 0.01). (J) Serum insulin levels before (0 min) and 15 min after oral glucose administration (*n* = 5, *p < 0.05, ***p < 0.001). (K) Co‐immunofluorescence staining of glucagon and insulin in pancreatic sections. Scale bar, 50 µm (*n* = 5, **p < 0.01). (L) Blood glucose curves during OGTTs and corresponding area under the curve (AUC) (*n* = 5, **p < 0.01). (M) Food intake analysis of WT and *Mstn^−/−^* mice (*n* = 5, *p < 0.05). (N) Schematic diagram of primary small intestinal organoid establishment from WT and *Mstn^−/−^* mice. (O) Representative images of primary small intestinal organoids derived from WT and *Mstn^−/−^* mice cultured for 5 days. (P) Representative images of GLP‐1 immunofluorescence staining in primary small intestinal organoids derived from WT and *Mstn^−/−^* mice Scale bar, 50 µm. Quantification of GLP‐1‐positive cells was based on 40 organoids from the WT group and 30 organoids from the *Mstn^−/−^* group (***p < 0.001). (Q–S), Active GLP‐1 levels in organoids derived from WT and *Mstn^−/−^* mice (*n* = 6, *p < 0.05, ***p < 0.001). GLP‐1 secretion was measured under basal conditions (Q), glucose stimulation (R), and forskolin/IBMX (F/I) stimulation (S). (T) Relative mRNA expression of *Pcsk1*, *Gcg*, *Gip*, and *Dpp4* in organoids (*n* = 6, *p < 0.05, **p < 0.01). Data are presented as mean ± s.e.m. *p*‐values were determined by two‐sided unpaired Student's t‐test (C, E, G, H, I, J, K, M, Q–S, the AUC analysis in L, and the *Pcsk1* analysis in T), two‐way repeated‐measures ANOVA followed by Tukey's post hoc test (L), or two‐sided Mann‐Whitney test (P and the *Gcg* analysis in T). *p < 0.05 was considered statistically significant.

Because pancreatic α cells may contribute to GLP‐1 production under selected pathophysiological conditions [19, 20], we examined pancreatic endocrine cell composition. α‐cell area was comparable between WT and *Mstn^−/−^* mice, whereas *Mstn^−/−^* mice displayed an enlarged β‐cell area (Figure 1K). We then assessed oral glucose tolerance, as GLP‐1 contributes to glycemic control by potentiating glucose‐stimulated insulin release and limiting postprandial glucose excursions. *Mstn^−/−^* mice exhibited improved oral glucose tolerance, accompanied by restricted body weight gain and reduced food intake (Figure 1L,M; Figure S1F). These findings suggest that the augmented insulin response observed in *Mstn^−/−^* mice may be coupled to increased GLP‐1 release after glucose challenge.

Beyond its glucoregulatory actions, GLP‐1 has been recognized as an important factor in maintaining intestinal barrier integrity [21, 22]. We therefore examined the expression of intestinal barrier‐associated proteins. In *Mstn^−/−^* mice, ileal ZO‐1 and Occludin expression was markedly increased and displayed a more continuous and intact membrane distribution pattern (Figure S1G,H), suggesting that MSTN deficiency is accompanied by improved intestinal junctional organization. We next asked whether the metabolic phenotype associated with MSTN deficiency was sex dependent. In HFD‐fed female mice, MSTN deletion similarly increased circulating active GLP‐1 and insulin levels after glucose stimulation compared with WT females (Figure S1I,J). Female *Mstn^−/−^* mice also showed increased pancreatic β‐cell area and improved oral glucose tolerance (Figure S1K,L). Overall, these findings consistently demonstrate that MSTN deficiency enhances intestinal GLP‐1 production and secretory capacity, accompanied by an augmented glucose‐stimulated insulin response.

Intestinal organoids are three‐dimensional mini‐gut models that preserve key epithelial cell lineages and architectural features in vitro [23]. To further determine the effect of MSTN deficiency on GLP‐1 secretion, we established small intestinal organoids derived from WT and *Mstn^−/−^* mice (Figure 1N,O). The proportion of GLP‐1^+^ cells was significantly increased in *Mstn^−/−^* organoids (Figure 1P). Under basal conditions, glucose stimulation, and combined forskolin/IBMX stimulation, *Mstn^−/−^* organoids consistently exhibited enhanced GLP‐1 secretory capacity (Figure 1Q–S). Meanwhile, the expression of GLP‐1 biosynthesis‐related genes *Gcg* and *Pcsk1* was upregulated, whereas *Gip* and *Dpp4* expression remained unchanged (Figure 1T). These results indicate that MSTN deficiency enhances both GLP‐1‐producing cell abundance and the secretory capacity of the intestinal epithelium.

### 2.2 Intestinal MSTN Deficiency Enhances GLP‐1 Secretion and Improves Glucose Homeostasis

Because global MSTN deficiency profoundly affects skeletal muscle and whole‐body metabolism [24], we sought to determine whether the enhanced GLP‐1 secretion in *Mstn^−/−^* mice was driven by loss of MSTN within the intestinal epithelium or by secondary systemic adaptations. To distinguish between these possibilities, we generated mice with intestinal epithelial cell‐specific deletion of *Mstn* (*Mstn^ΔIE^*) (Figure 2A–C; Figure S2A). We confirmed the knockout of MSTN in the intestine (Figure S2B), and MSTN expression in other tissues was unaffected (Figure S2C). Next, we fed *Mstn^fl/fl^* and *Mstn^ΔIE^* mice an HFD for 12 weeks (Figure 2D). Consistent with the phenotype observed in the global knockout mice, *Mstn^ΔIE^* mice showed an increased number of intestinal GLP‐1^+^ cells after HFD feeding (Figure 2E). Ileal expression of the GLP‐1 biosynthesis‐related genes *Gcg* and *Pcsk1* was also increased, whereas *Gip* and *Dpp4* expression was not markedly changed (Figure 2F). Consistent with these transcriptional changes, the protein levels of GCG and PC1/3 were also significantly increased (Figure S2B), suggesting that intestinal epithelial MSTN deficiency is sufficient to enhance GLP‐1 biosynthetic capacity. In line with this, *Mstn^ΔIE^* mice exhibited higher circulating active GLP‐1 and insulin levels even under basal conditions, and these differences were further amplified after glucose stimulation (Figure 2G,H). In addition, fasting blood glucose was significantly reduced in *Mstn^ΔIE^* mice compared with *Mstn^fl/fl^* mice (Figure S2D). To further verify whether the improvement in glucose metabolism associated with MSTN deficiency was related to the action of GLP‐1, we performed a blocking experiment using the GLP‐1R antagonist Exendin(9‐39). After Ex9‐39 treatment, the increased of insulin and improved glucose tolerance phenotype in *Mstn^ΔIE^* mice was markedly attenuated, indicating that GLP‐1 action is involved in the improvement of glucose metabolism associated with MSTN deficiency (Figure 2I). In addition to improved glucose handling, *Mstn^ΔIE^* gained less body weight during HFD feeding and showed reduced food intake (Figure 2J,K). Pancreatic α‐cell area was comparable between groups, whereas β‐cell area was greater in *Mstn^ΔIE^* mice (Figure 2L; Figure S2E). We also assessed tight‐junction‐associated proteins in the ileum. ZO‐1 and Occludin expression were increased in *Mstn^ΔIE^* mice (Figure 2M,N), indicating that intestinal epithelial MSTN deficiency is accompanied by a more favorable junctional phenotype. In addition, we analyzed whether intestinal epithelial MSTN deficiency altered whole‐body energy metabolism. The temporal profiles of VO_2_, VCO_2_, respiratory exchange ratio, and heat production were largely overlapping between *Mstn^ΔIE^* and *Mstn^fl/fl^* mice. Quantitative analysis of the average values during both light and dark cycles revealed no genotype‐dependent shift in these metabolic parameters (Figure 2O,P; Figure S2F,G). Together with the global knockout data, these findings show that intestinal epithelial MSTN deficiency is sufficient to enhance GLP‐1 production and secretion, strengthen glucose‐stimulated insulin responses, and improve glucose tolerance under HFD conditions.

**FIGURE 2:**
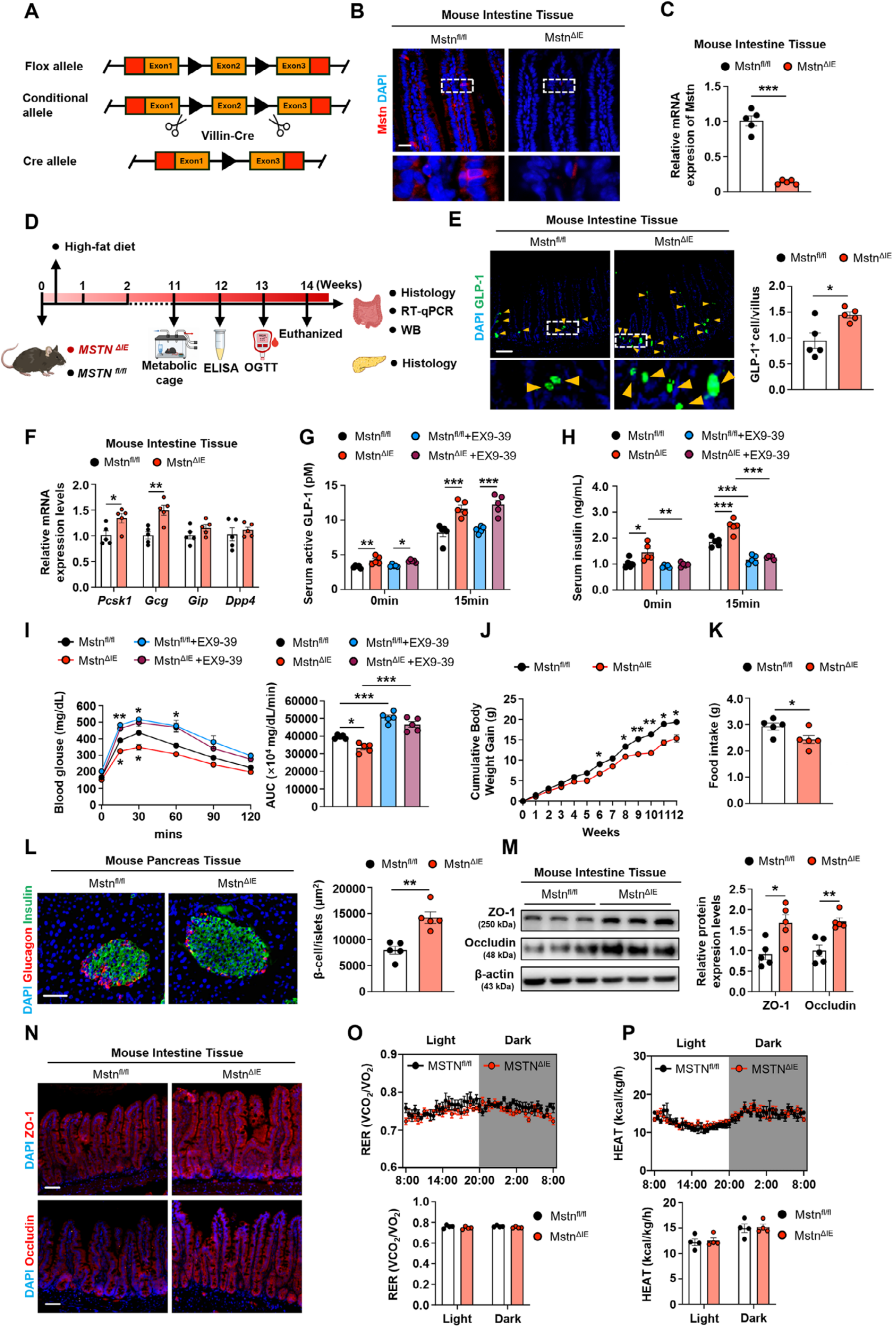
Intestinal epithelial MSTN deficiency enhances GLP‐1 secretion. (A) Schematic diagram illustrating the generation of intestinal epithelium‐specific *Mstn* knockout (*Mstn^ΔIE^*) mice. (B) Immunohistochemistry of MSTN expression in the small intestine of *Mstn^fl/fl^* and *Mstn^ΔIE^* mice (*n* = 5). (C) *Mstn* mRNA expression in the small intestine of *Mstn^fl/fl^* and *Mstn^ΔIE^* mice (*n* = 5, ***p < 0.001). (D) Schematic diagram of the experimental design for HFD feeding in *Mstn^fl/fl^* and *Mstn^ΔIE^* mice. (E) Representative images of GLP‐1 immunofluorescence staining in small intestine sections. Scale bar, 50 µm (*n* = 5, *p < 0.05). (F) Relative mRNA expression of *Pcsk1*, *Gcg*, *Gip*, and *Dpp4* in the small intestine (*n* = 5, *p < 0.05, **p < 0.01). (G, H) Serum active GLP‐1 (G) and insulin (H) levels before (0 min) and 15 min after oral glucose administration (*n* = 5, *p < 0.05, **p < 0.01). (I) Blood glucose curves during OGTTs and corresponding AUC (*n* = 5, *p < 0.05, **p < 0.01, ***p < 0.001). (J, K) Cumulative body weight gain (J), food intake (K) analysis of *Mstn^fl/fl^* and *Mstn^ΔIE^* mice (*n* = 5, *p < 0.05, **p < 0.01). (L) Co‐immunofluorescence staining of Glucagon and Insulin in pancreatic sections. Scale bar, 50 µm (*n* = 5, **p < 0.01). (M) Expression of the tight junction proteins ZO‐1 and Occludin in the small intestine (*n* = 5, *p < 0.05, **p < 0.01). (N) Representative images of tight junction proteins immunofluorescence staining in small intestine sections. Scale bar, 50 µm (*n* = 5). O, P, respiratory exchange ratio (RER) (O), and average energy expenditure (P) analysis (*n* = 4). Data are presented as mean ± s.e.m. *p*‐values were determined by two‐sided unpaired Student's t‐test (C, E, F, K–M, O and P), one‐way ANOVA followed by Tukey's post hoc test (the AUC analysis in I), or two‐way repeated‐measures ANOVA followed by Tukey's post hoc test (I and J). *p < 0.05 was considered statistically significant.

We next evaluated whether this regulatory effect was dependent on metabolic stress. Under normal chow conditions, *Mstn^ΔIE^* mice showed no obvious differences in body weight, cumulative body weight gain, or oral glucose tolerance compared with *Mstn^fl/fl^* controls (Figure S2H,I). Nevertheless, ileal Gcg and Pcsk1 expression remained increased in *Mstn^ΔIE^* mice accompanied by higher circulating active GLP‐1 and insulin levels after glucose stimulation (Figure S2J–L). Thus, intestinal MSTN deficiency persistently enhances GLP‐1 production, whereas its impact on whole‐body glucose metabolism becomes more evident under metabolic stress.

### 2.3 Intestinal MSTN Regulates the Membrane Enrichment of SGLT1

The selective increase in glucose‐stimulated GLP‐1 secretion raised the possibility that MSTN deficiency may not uniformly amplify enteroendocrine responses to all nutrient cues. We therefore challenged mice with corn oil, a lipid stimulus for GLP‐1 release. In contrast to the glucose challenge, the corn oil‐evoked GLP‐1 response was largely unchanged after either intestinal epithelial or global *Mstn* deletion (Figure 3A,B). This nutrient‐specific pattern suggested that MSTN preferentially modulates the glucose‐responsive arm of GLP‐1 secretion. Given the central role of SGLT1 and GLUT2 in intestinal glucose sensing [25, 26], we assessed *Slc5a1* and *Slc2a2* expression. Neither transcript was appreciably altered in MSTN‐deficient mice (Figure 3C,D). However, immunofluorescence analysis showed enhanced SGLT1 signaling with no obvious change in GLUT2 signaling in both knockout models (Figure S3A). To determine whether MSTN deficiency promotes membrane localization of SGLT1 in L cells in vivo, we analyzed the localization relationship between GLP‐1^+^ L cells and SGLT1 in ileal tissue by immunofluorescence co‐staining. The results showed that, compared with control mice, membrane‐surface SGLT1 signaling in GLP‐1^+^ L cells was markedly enhanced in MSTN‐deficient mice (Figure 3E). This discrepancy between unchanged *Slc5a1* transcription and increased apical SGLT1 protein suggested that MSTN may regulate glucose sensing by controlling SGLT1 membrane availability. To test this possibility, we separated cytosolic and membrane fractions from small intestinal tissue. In both *Mstn^ΔIE^* and *Mstn^−/−^*, SGLT1 abundance was selectively increased in the membrane fraction, with little change in the cytosolic pool (Figure 3F; Figure S3B).

**FIGURE 3:**
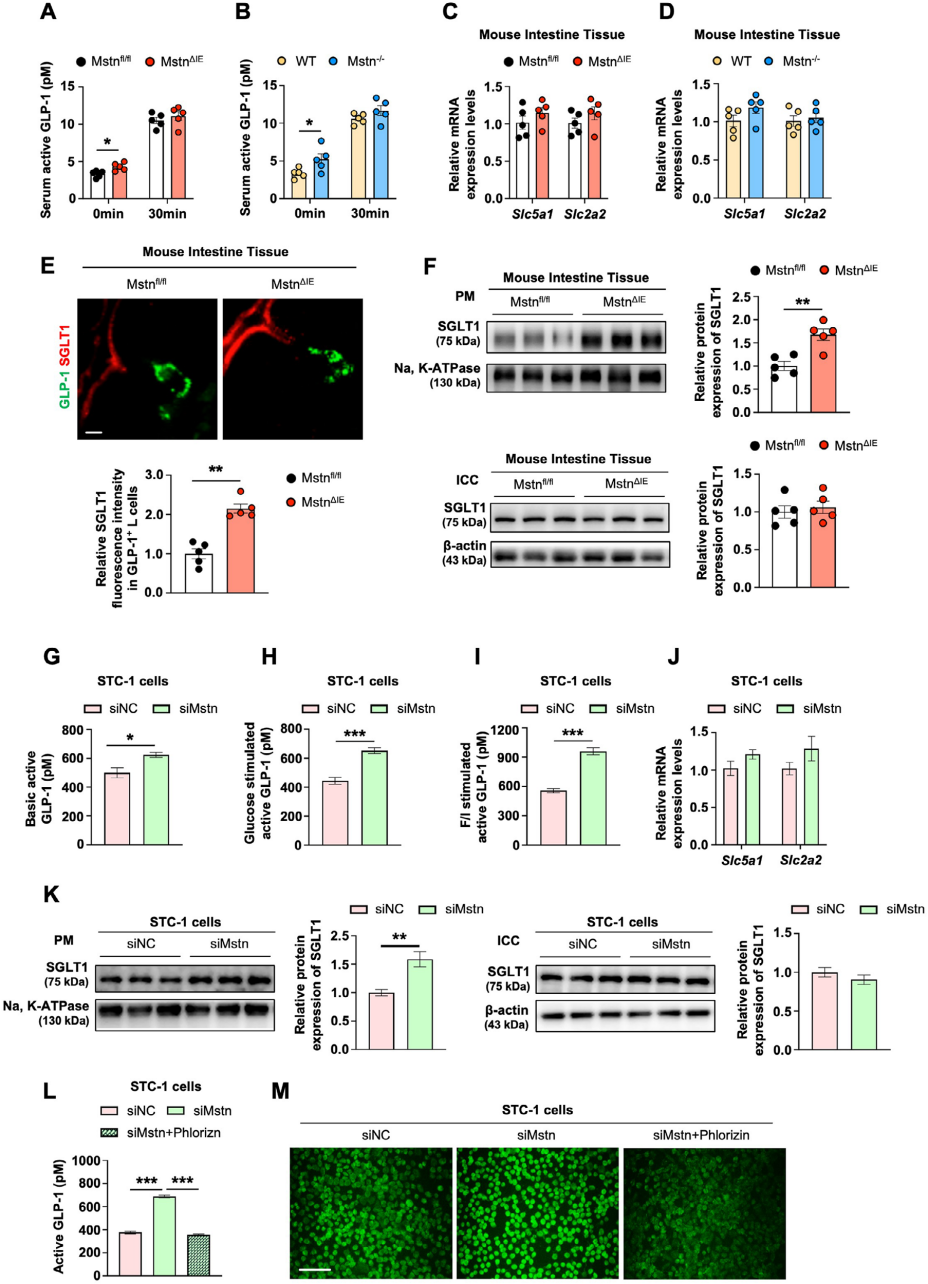
Intestinal epithelial deletion of MSTN increases plasma membrane abundance of SGLT1. (A, B) Serum active GLP‐1 levels before (0 min) and 30 min after oral corn oil administration in *Mstn^fl/fl^* and *Mstn^ΔIE^* mice (A, *n* = 5, *p < 0.05) and WT and *Mstn^−/−^* mice (B, *n* = 5, *p < 0.05). (C, D) Relative mRNA expression of *Slc5a1* and *Slc2a2* in the small intestine of *Mstn^fl/fl^* and *Mstn^ΔIE^* mice (C, *n* = 5) and WT and *Mstn^−/−^* mice (D, *n* = 5). (E) Representative images of immunofluorescence staining of membrane‐surface SGLT1 signaling in GLP‐1^+^ L cells in the small intestine sections from *Mstn^fl/fl^* and *Mstn^ΔIE^* mice. Scale bar, 5 µm (*n* = 5). (F) Immunoblot analysis of SGLT1 in plasma membrane and cytosolic fractions isolated from small intestine tissues (**p < 0.01). (G–I) Active GLP‐1 levels in the culture supernatants of STC‐1 cells after siMstn transfection (*n* = 6, *p < 0.05, ***p < 0.001). GLP‐1 secretion was measured under basal conditions (G), glucose stimulation (H), and forskolin/IBMX stimulation (I). (J) Relative mRNA expression of *Slc5a1* and *Slc2a2* in STC‐1 cells (*n* = 6). (K) Immunoblot analysis of SGLT1 in plasma membrane and cytosolic fractions isolated from STC‐1 cells transfected with siNC or siMstn (*n* = 6, **p < 0.01). (L) Active GLP‐1 levels in culture supernatants following treatment with 100 µM phlorizin (*n* = 6, ***p < 0.001). (M) 2‐NBDG uptake following treatment with 100 µM phlorizin (*n* = 6). Scale bar, 50 µm. Data are presented as mean ± s.e.m. *p* values were determined by two‐sided unpaired Student's t‐test (A–E, F–K) or one‐way ANOVA followed by Tukey's post hoc test (L). *p < 0.05 was considered statistically significant.

We then tested this regulatory pattern in STC‐1 enteroendocrine cells. MSTN silencing increased GLP‐1 secretion under basal conditions, after glucose stimulation, and in response to combined forskolin/IBMX treatment (Figure 3G–I). It also increased GCG and PC1/3 protein abundance, together with higher *Gcg* and *Pcsk1* expression (Figure S3C,D). As observed in vivo findings, *Mstn* silencing did not alter *Slc5a1* or *Slc2a2* mRNA expression in STC‐1 cells (Figure 3J). Instead, SGLT1 protein was preferentially enriched at the cell membrane, with no appreciable increase in the cytoplasmic fraction (Figure 3K). To determine whether SGLT1‐dependent glucose uptake was required for the effect of *Mstn* inhibition, we treated STC‐1 cells with phlorizin, an SGLT inhibitor, and assessed 2‐NBDG uptake and GLP‐1 secretion. Phlorizin abolished the increase in glucose uptake and GLP‐1 secretion induced by *Mstn* silencing (Figure 3L,M). These findings place SGLT1 membrane enrichment and SGLT1‐dependent glucose uptake downstream of intestinal MSTN in the control of GLP‐1 release.

### 2.4 SGLT1 S‐Palmitoylation at Cys301 Maintains Its Plasma Membrane Localization

We next investigated the mechanism by which SGLT1 accumulates at the cell surface. Palmitoylation regulates protein trafficking, membrane association, and subcellular distribution, prompting us to examine whether this lipid modification contributes to SGLT1 membrane enrichment [27]. We therefore explored whether protein palmitoylation mediates the increased membrane localization of SGLT1. Using acyl‐biotin exchange assays (ABE), we detected a clear palmitoylation signal for SGLT1 that was dependent on hydroxylamine treatment [28], consistent with the presence of a reversible thioester‐linked lipid modification (Figure 4A). To further validate the lipid‐modified state of SGLT1, we performed metabolic labelling with the biorthogonal palmitate probe Alk‐C16. Both endogenous and ectopically expressed SGLT1 were efficiently labelled by Alk‐C16 (Figure 4B,C), supporting that SGLT1 undergoes C16 fatty acylation/palmitoylation. Notably, *Mstn* silencing increased SGLT1 palmitoylation, an effect that was reversed by 2‐bromopalmitate (2‐BP) (Figure 4D). Pharmacological inhibition of palmitoylation also reduced the functional effects induced by *Mstn* silencing. 2‐BP attenuated the increase in SGLT1 plasma membrane localization, GLP‐1 biosynthesis and secretion, and 2‐NBDG uptake caused by *Mstn* inhibition (Figure 4E–G; Figure S4A). In addition, SGLT1 overexpression increased the abundance of GCG and its processing enzyme PC1/3, whereas 2‐BP treatment markedly blunted this effect (Figure 4H). These results indicate that palmitoylation is required for SGLT1 membrane enrichment and for the downstream pro‐secretory effects associated with *Mstn* inhibition.

**FIGURE 4:**
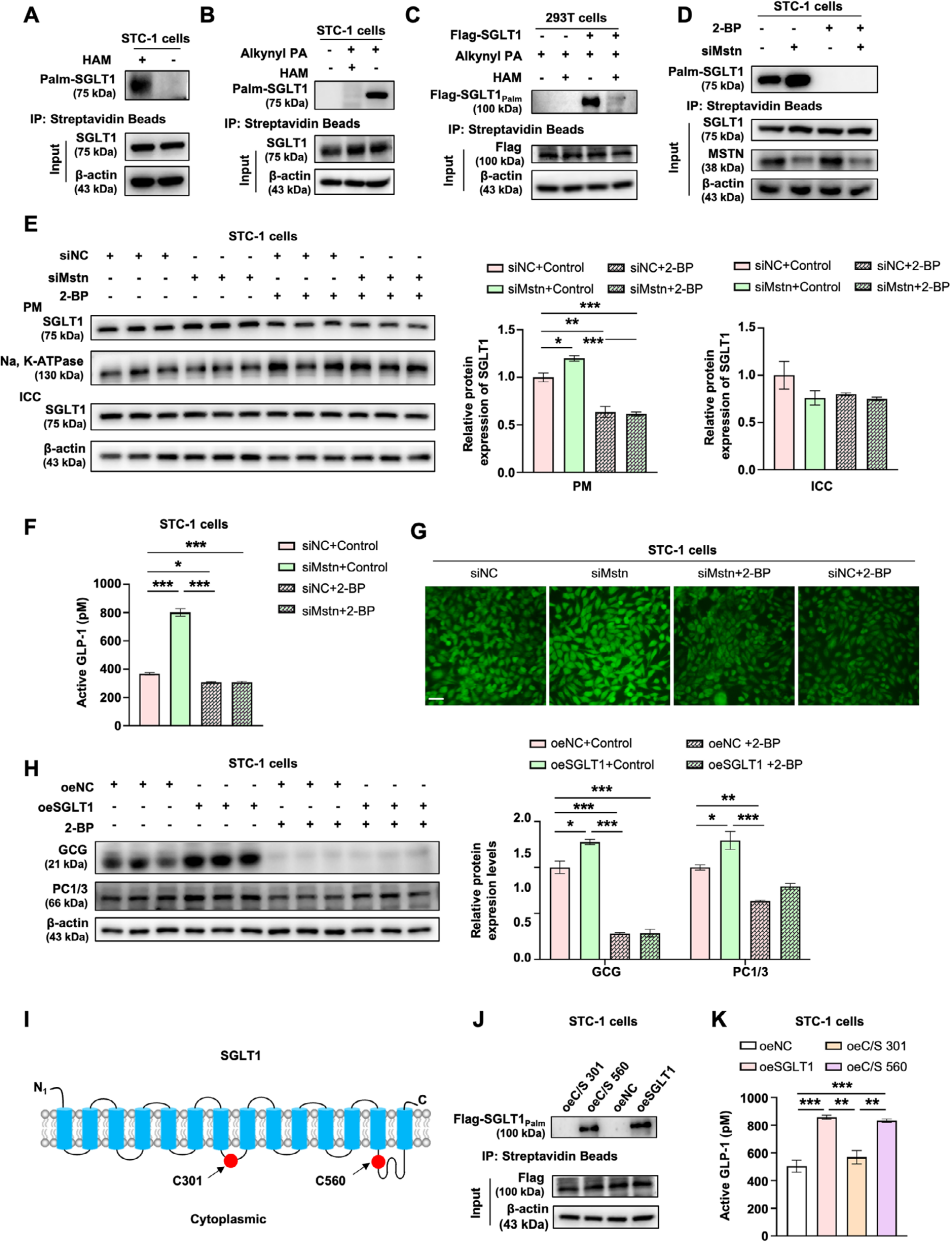
Palmitoylation at Cys301 promotes SGLT1 enrichment at the plasma membrane. (A) S‐palmitoylation levels of endogenous SGLT1 in STC‐1 cells with hydroxylamine (HA+) or without (HA‐) treatment, analyzed by the acyl‐biotin exchange (ABE) assay. (B) S‐palmitoylation levels of SGLT1 in STC‐1 cells analyzed by click chemistry. (C) S‐palmitoylation levels of exogenous SGLT1 in HEK293T cells overexpressing Flag‐SGLT1, analyzed by the ABE assay. (D) S‐palmitoylation levels of SGLT1 in STC‐1 cells transfected with siNC or siMstn following 2‐BP (50 µM) treatment. (E) Immunoblot analysis of SGLT1 protein levels in plasma membrane and cytosolic fractions of STC‐1 cells transfected with siNC or siMstn following 2‐BP (50 µM) treatment (*n* = 3, *p < 0.05, **p < 0.01, ***p < 0.001). (F) Active GLP‐1 levels in the culture supernatants of STC‐1 cells transfected with siNC or siMstn following 2‐BP (50 µM) treatment (*n* = 6, *p < 0.05, ***p < 0.001). (G) 2‐NBDG uptake levels in STC‐1 cells transfected with siNC or siMstn following 2‐BP (50 µM) treatment. Scale bar, 50 µm (*n* = 6). (H) Immunoblot analysis of GCG and PC1/3 protein levels in STC‐1 cells overexpressing SGLT1 (*n* = 3, *p < 0.05, **p < 0.01, ***p < 0.001). (I) Schematic diagram of SGLT1 protein membrane topology and candidate palmitoylation sites. (J) S‐palmitoylation levels of SGLT1 in STC‐1 cells overexpressing WT Flag‐SGLT1, C301S, or C560S mutants, analyzed by the ABE assay. (K) Active GLP‐1 levels in the culture supernatants of STC‐1 cells transfected with WT SGLT1, C301S, or C560S mutants (*n* = 3, **p < 0.01, ***p < 0.001). Data are presented as mean ± s.e.m. *p*‐values were determined by one‐way ANOVA followed by Tukey's post hoc test (E, F, G, K). *p < 0.05 was considered statistically significant.

Because palmitoylation of transmembrane proteins usually occurs on cytosol‐facing cysteine residues [29, 30], we next sought to identify the potential palmitoylation site of SGLT1. Based on the predicted transmembrane topology of SGLT1 from UniProt and PDBTM, we selected two evolutionarily conserved cytosol‐facing cysteine residues, Cys301 and Cys560, as candidate palmitoylation sites (Figure 4I; Figure S4B). After site‐directed mutagenesis of these two residues, ABE analysis showed that the palmitoylation signal was markedly reduced in the SGLT1‐C301S mutant, whereas the SGLT1‐C560S mutation had little effect (Figure 4J). Furthermore, overexpression of SGLT1‐C301S failed to increase secreted GLP‐1 levels, GLP‐1 biosynthesis, or 2‐NBDG uptake. In contrast, SGLT1‐C560S retained pro‐secretory and glucose uptake‐promoting effects comparable to those of wild‐type SGLT1 (Figure 4K; Figure S4C,D). Together, these results collectively identify Cys301 as a critical palmitoylation site required for SGLT1 membrane localization and its associated functional effects.

### 2.5 ZDHHC20 Mediates SGLT1 S‐Palmitoylation

Protein S‐palmitoylation is installed by DHHC‐domain‐containing acyltransferases, a 23‐member enzyme family with distinct tissue distribution, subcellular localization, and substrate preference [30]. To identify the enzyme linking MSTN signaling to SGLT1 palmitoylation, we mined the Human Protein Atlas for intestinally expressed ZDHHC enzymes and prioritized candidates whose annotated subcellular localization was compatible with SGLT1 distribution, including cytoplasmic and plasma membrane compartments. Although most *Zdhhc* members showed basal expression in the intestine, *Zdhhc20* was markedly upregulated upon *Mstn* inhibition (Figure S5A,B). Consistently, *Zdhhc20* expression was significantly increased in the intestine of both *Mstn^−/−^* and *Mstn^ΔIE^* mice (Figure 5A,B), suggesting that ZDHHC20 may participate in MSTN‐regulated SGLT1 modification. Structural prediction and molecular docking analysis suggested a potential interaction interface between ZDHHC20 and SGLT1 (Figure 5C). To validate whether SGLT1 interacts with ZDHHC20, Flag‐tagged SGLT1 and HA‐tagged Zdhhc20 were co‐transfected into STC‐1 cells, followed by co‐immunoprecipitation (Co‐IP) analysis. HA‐Zdhhc20 was clearly enriched after immunoprecipitation with an anti‐Flag antibody. Conversely, Flag‐SGLT1 was also detected after immunoprecipitation with an anti‐HA antibody (Figure 5D), indicating that SGLT1 and ZDHHC20 exist in the same protein complex in cells.

**FIGURE 5:**
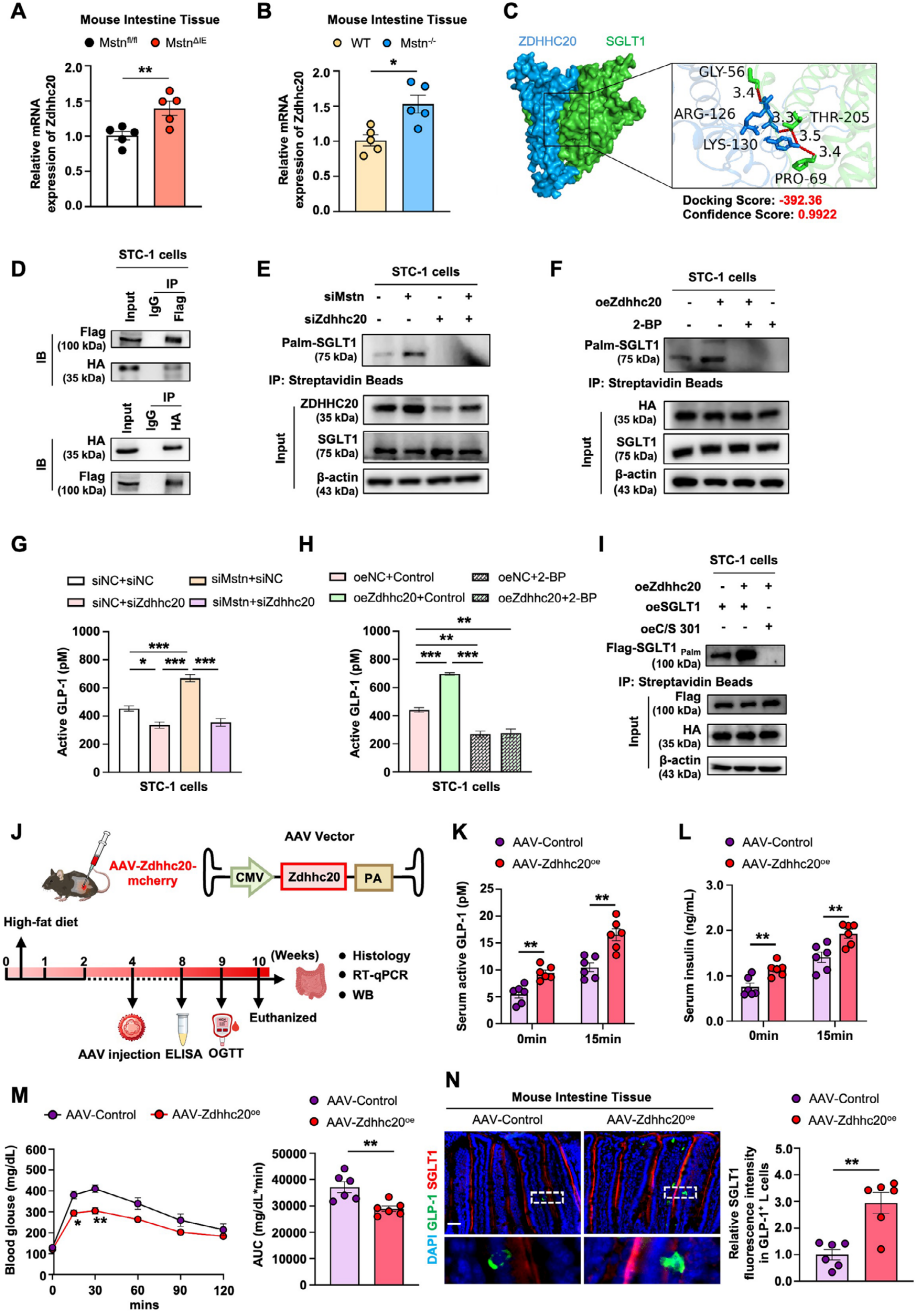
Palmitoylation of SGLT1 depends on ZDHHC20. (A, B) Relative mRNA expression of *Zdhhc20* in the ileum of *Mstn^fl/fl^* and *Mstn^ΔIE^* mice (A, *n* = 5, **p < 0.01) and WT and *Mstn^−/−^* mice (B, *n* = 5 mice per group, *p < 0.05) fed a HFD for 12 weeks. (C) Schematic illustration of the molecular docking between ZDHHC20 and SGLT1. (D) Co‐IP analysis of cell lysates from STC‐1 cells co‐transfected with HA‐Zdhhc20 and Flag‐SGLT1. (E) S‐palmitoylation levels of SGLT1 in STC‐1 cells transfected with siNC, siMstn, or siZdhhc20, analyzed by the ABE assay. (F) S‐palmitoylation levels of SGLT1 in STC‐1 cells overexpressing Zdhhc20 (oeZdhhc20), analyzed by the ABE assay. (G, H) Active GLP‐1 levels in the culture supernatants of STC‐1 cells under the indicated treatment conditions (*n* = 3, *p < 0.05, **p < 0.01, ***p < 0.001). (I) S‐palmitoylation levels of SGLT1 under the indicated treatment conditions analyzed by the ABE assay. (J) Schematic diagram of the experimental workflow for generating the intestinal Zdhhc20‐overexpressing (AAV‐Zdhhc20^oe^) mouse model. (K, L) Serum active GLP‐1 (K) and insulin (L) levels before (0 min) and 15 min after oral glucose administration (*n* = 6, **p < 0.01). (M) Blood glucose curves during OGTTs and corresponding AUC (*n* = 6, *p < 0.05, **p < 0.01). (N) Immunofluorescence staining of membrane‐surface SGLT1 signaling in GLP‐1^+^ L cells in ileal sections. Scale bar, 50 µm (*n* = 6). Data are presented as mean ± s.e.m. *p*‐values were determined by two‐sided unpaired Student's t‐test (A, B, K, L and the AUC analysis in M), one‐way ANOVA followed by Tukey's post hoc test (G, H), or two‐way repeated‐measures ANOVA followed by Tukey's post hoc test (M). *p < 0.05 was considered statistically significant.

We next silenced *Zdhhc20* expression in STC‐1 cells. *Zdhhc20* knockdown significantly reduced *Gcg* and *Pcsk1* expression and suppressed GCG and PC1/3 protein abundance (Figure S5C,E). Crucially, *Zdhhc20* knockdown blunted the increase in SGLT1 palmitoylation and GLP‐1 secretion induced by *Mstn* inhibition (Figure 5E,G). Conversely, *Zdhhc20* overexpression upregulated GLP‐1 biosynthesis‐related genes and protein levels (Figure S5D,F), and enhanced SGLT1 palmitoylation, GLP‐1 secretion, and 2‐NBDG uptake (Figure 5F,H; Figure S5G). These effects were partially reversed by 2‐BP, supporting their dependence on palmitoylation. Obviously, *Zdhhc20* overexpression had little effect on the residual palmitoylation signal of the SGLT1‐C301S mutant (Figure 5I).

To determine whether increased *Zdhhc20* expression contributes to glucose tolerance in vivo, we used an HBAAV2/9‐mediated intestinal epithelial‐targeted delivery strategy [31, 32] to induce intestinal *Zdhhc20* overexpression in HFD‐fed mice (Figure 5J; Figure S6A,B). The results showed that *Zdhhc20* overexpression significantly enhanced active GLP‐1 release and insulin secretion under both basal and glucose‐stimulated conditions (Figure 5K,L). Strikingly, mice with intestinal *Zdhhc20* overexpression showed improved oral glucose tolerance (Figure 5M). At the intestinal level, *Zdhhc20* markedly enhanced membrane‐surface SGLT1 signaling in GLP‐1^+^ L cells and increased intestinal GCG and PC1/3 protein abundance (Figure 5N; Figure S6C). Together, these data reveal that ZDHHC20 as a major acyltransferase catalyzing SGLT1 palmitoylation and demonstrate its role in regulating intestinal GLP‐1 secretion and glucose homeostasis.

### 2.6 MSTN Inhibition Promotes YY1‐Dependent *Zdhhc20* Transcription

Given that MSTN deficiency or inhibition increased *Zdhhc20* mRNA expression, we speculated that MSTN may regulate *Zdhhc20* transcription through specific transcription factors. We performed transcriptomic sequencing of intestinal tissues from MSTN‐KO, which revealed significant upregulation of the transcription factor YY1 (SRA: PRJNA1531303). Previous studies have shown that YY1 participates in intestinal villus development and epithelial differentiation, and that YY1 deficiency leads to abnormal intestinal differentiation and defective villus development [33, 34]. To determine whether YY1 could serve as a potential transcriptional regulator of *Zdhhc20*, we further mined publicly available intestinal transcriptomic datasets, including GSE240206 and GSE288466. The analysis revealed a highly significant positive correlation between *Yy1* and *Zdhhc20* expression (Figure 6A), and this concordant expression pattern was further supported by analysis based on the TIMER 3.0 database (Figure 6B). Of note, transcription factor binding motif prediction combined with chromatin immunoprecipitation sequencing (ChIP‐seq) analysis showed a prominent YY1 enrichment signal in the *Zdhhc20* promoter region, which overlapped well with the predicted YY1 binding motif (Figure 6C,D). These findings suggest that YY1 may act as a transcriptional activator of Zdhhc20 and mediate MSTN‐dependent transcriptional regulation of ZDHHC20 expression.

**FIGURE 6:**
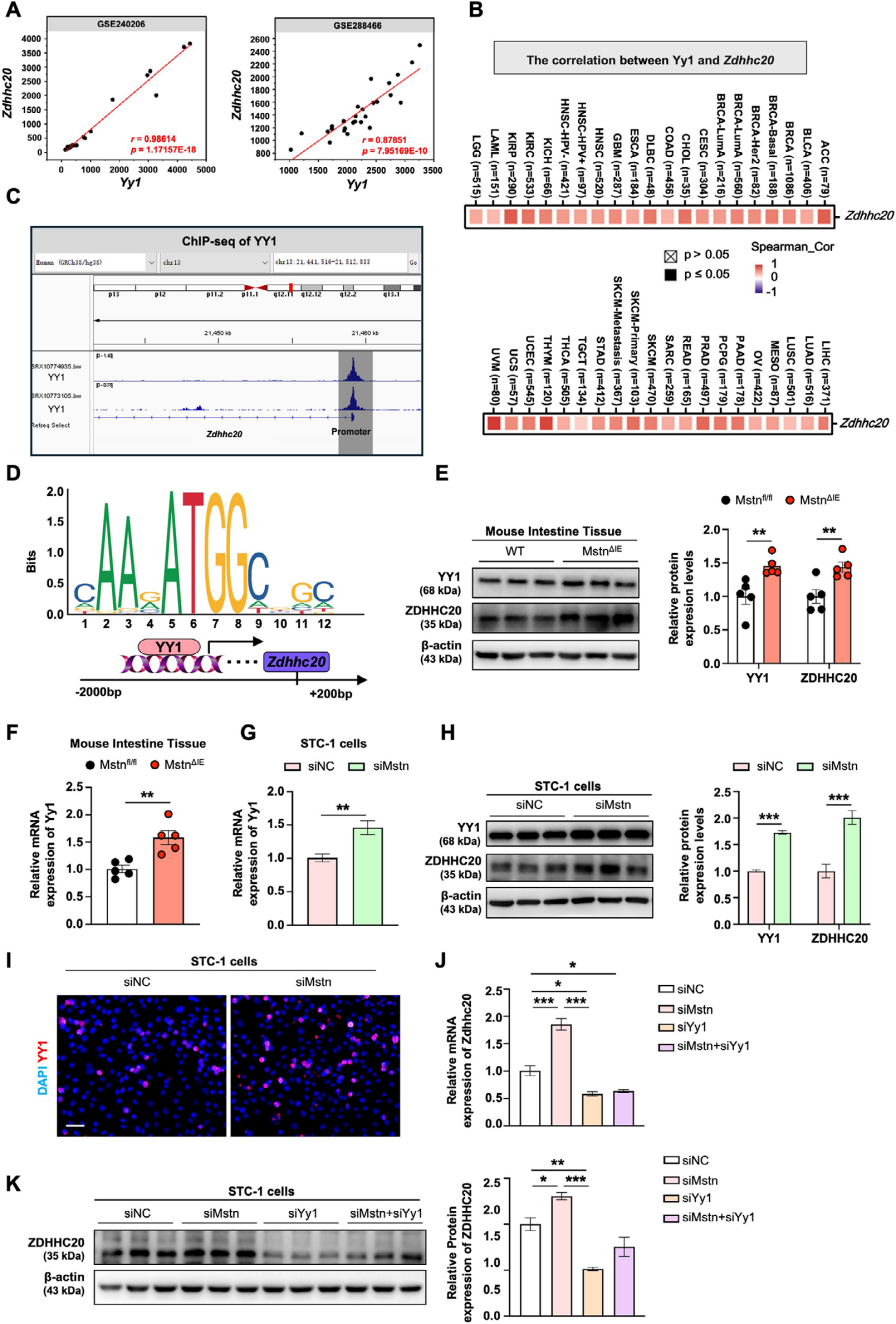
MSTN inhibition upregulates ZDHHC20 expression through transcription factor YY1. (A) Spearman correlation analysis between *Yy1* and *Zdhhc20* expression in the GSE240206 and GSE288466 datasets. (B) Correlation heatmap showing the association between *Yy1* and *Zdhhc20* expression generated using TIMER 3.0. (C) ChIP‐Atlas analysis showing YY1 occupancy at the *Zdhhc20* promoter region. (D) Predicted YY1‐binding motif with in the *Zdhhc20* promoter region. (E) Immunoblot analysis of YY1 and ZDHHC20 protein levels in ileal tissues (*n* = 5, **p < 0.01). (F) Relative *Yy1* mRNA expression in WT and *Mstn^−/−^* mice (*n* = 5, **p < 0.01). (G, H) STC‐1 cells transfected with siNC or siMstn (*n* = 6, **p < 0.01, ***p < 0.001). Relative *Yy1* mRNA expression (G). Immunoblot analysis of YY1 and ZDHHC20 protein levels (H). (I) Immunofluorescence staining of YY1 in STC‐1 cells transfected with siNC or siMstn. Scale bar, 50 µm. (J) Expression of ZDHHC20 mRNA levels in STC‐1 cells after co‐transfection with siYy1 and siMSTN. (K) Immunoblot analysis of ZDHHC20 protein levels in STC‐1 cells after co‐transfection with siYy1 and siMSTN. (*n* = 3, *p < 0.05, ***p < 0.001). Data are presented as mean ± s.e.m. *p* values were determined by Spearman's rank correlation (A), two‐sided unpaired Student's t‐test (E–H), or one‐way ANOVA followed by Tukey's post hoc test (J, K). *p < 0.05 was considered statistically significant.

Subsequently, we examined YY1 expression in the intestine of *Mstn^−/−^* and *Mstn^ΔIE^* mice, as well as in siMstn‐treated STC‐1 cells. In all three models, YY1 and ZDHHC20 were coordinately upregulated at both the RNA and protein levels (Figure 6E–H; Figure S7A,B). *Mstn* silencing also markedly increased nuclear localization of YY1 in STC‐1 cells (Figure 6I). We further performed combined knockdown experiments of Mstn and Yy1 to clarify the role of YY1 in the regulation of ZDHHC20 by MSTN deficiency. The results showed that *Yy1* knockdown significantly attenuated the increase in ZDHHC20 expression induced by *Mstn* knockdown (Figure 6J,K, Figure S7C,D). Interestingly, in both GSE240206 and GSE288466 datasets, *Yy1* and *Zdhhc20* also showed concordant expression patterns with *Gcg* and *Pcsk1* (Figure S7E–H).

### 2.7 Simvastatin Upregulates MSTN Expression

Considering that simvastatin has been associated with an increased risk of blood glucose homeostasis dysregulation and has been reported to induce skeletal muscle atrophy accompanied by increased MSTN expression [7, 35, 36], we investigated whether MSTN deficiency could alleviate simvastatin‐induced impairment of glucose metabolism. We first evaluated the relationship between simvastatin and GLP‐1 secretion in intestinal enteroendocrine cells. Simvastatin reduced 2‐NBDG uptake in a dose‐dependent manner and concomitantly decreased GLP‐1 levels in the culture supernatant (Figure 7A,B). Remarkably, simvastatin treatment induced MSTN expression while suppressing GCG and PC1/3 expression (Figure 7C). We further examined this effect in vivo (Figure 7D). Oral administration of simvastatin (40 mg/kg/day) [7, 37, 38] induced intestinal MSTN upregulation and inhibited GLP‐1 synthesis and secretion. However, these effects were markedly attenuated in the context of MSTN deficiency (Figure 7E–G). Moreover, simvastatin impaired insulin secretion and glucose intolerance, accompanied by reduced SGLT1 distribution at the brush border of ileal villi. In contrast, MSTN deficiency largely reversed these changes (Figure 7H–J). Two‐way ANOVA revealed significant genotype × treatment interactions, indicating that the effects of simvastatin were significantly modified by MSTN genotype (Figure 7G,H). These results suggest that MSTN deficiency alleviates simvastatin‐induced impairment of glucose tolerance by preserving intestinal GLP‐1 secretion.

**FIGURE 7:**
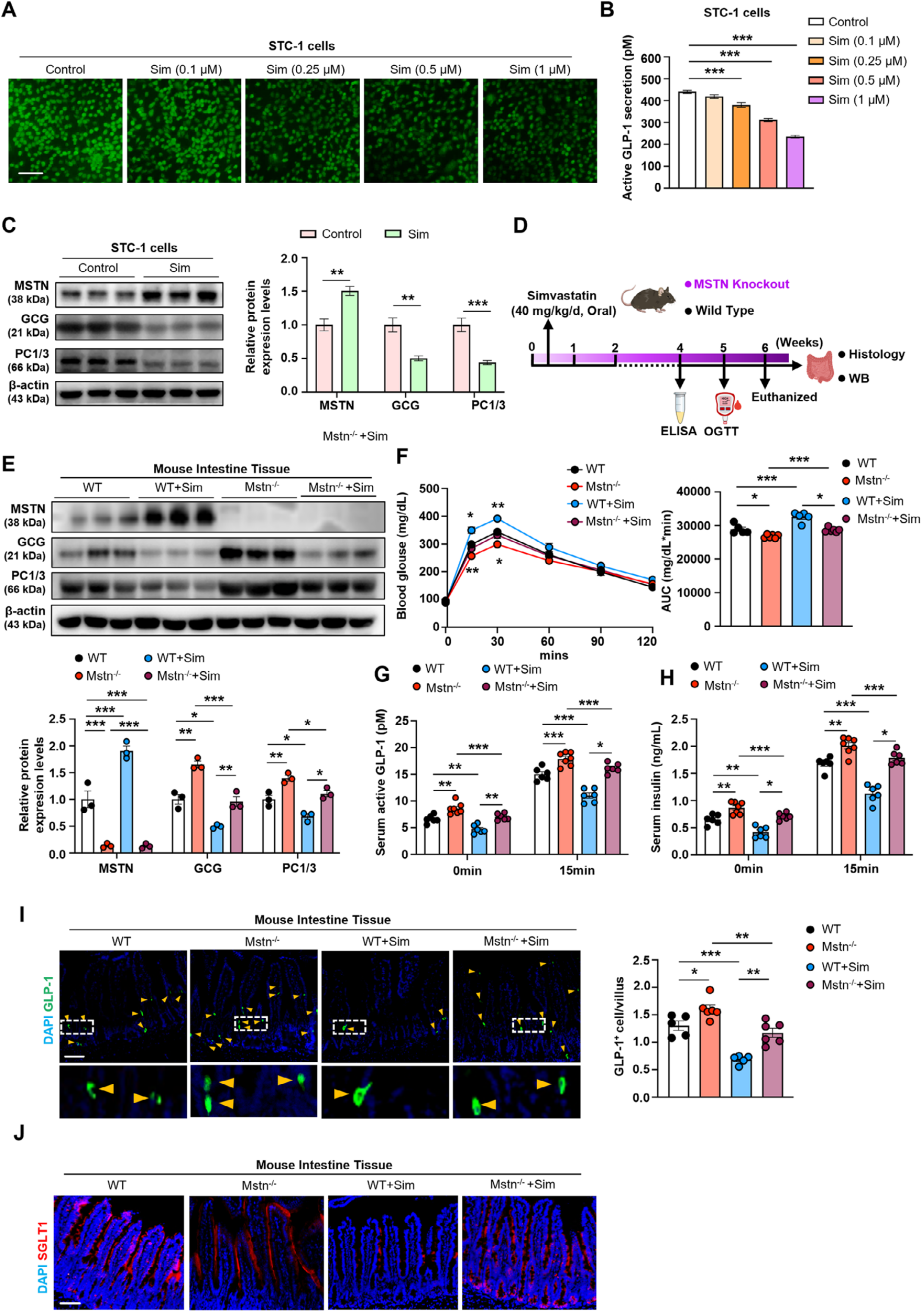
MSTN deficiency alleviates simvastatin‐induced glucose intolerance. (A, B) STC‐1 cells treated with the indicated concentrations of simvastatin (Sim). 2‐NBDG uptake (A) and active GLP‐1 secretion (B) were determined (*n* = 5, ***p < 0.001). (C) Immunoblot analysis of MSTN, GCG, and PC1/3 protein levels in STC‐1 cells following Sim treatment (*n* = 5, *p < 0.05, **p < 0.01, ***p < 0.001). (D) Schematic diagram of the experimental workflow for simvastatin treatment. (E) Immunoblot analysis of MSTN, GCG, and PC1/3 protein levels in ileal tissues. WT, WT + sim, *Mstn^−/−^* and *Mstn^−/−^* + sim mice (*n* = 3, *p < 0.05, **p < 0.01). (F) Oral glucose tolerance test (OGTT) and corresponding AUC. WT, WT + sim, *Mstn^−/−^* and *Mstn^−/−^* + sim mice (WT, *n* = 5 mice; WT + simvastatin, *n* = 5 mice; *Mstn^−/−^*, *n* = 6 mice; Mstn^−/−^ + simvastatin, *n* = 6 mice, *p < 0.05, ***p < 0.001). (G) Serum active GLP‐1 levels at 0 and 15 min following oral glucose administration. Two‐way ANOVA: Genotype, *p* < 0.001; treatment interaction, *p* < 0.001; genotype × treatment interaction, *p* < 0.01. WT, WT + sim, *Mstn^−/−^* and *Mstn^−/−^* + sim mice (WT, *n* = 6 mice; WT + simvastatin, *n* = 6 mice; *Mstn^−/−^*, *n* = 7 mice; *Mstn^−/−^* + simvastatin, *n* = 6 mice, *p < 0.05, **p < 0.01, ***p < 0.001). (H) Serum insulin levels following oral glucose administration. Two‐way ANOVA: Genotype, *p* < 0.001; treatment interaction, *p* < 0.001; genotype × treatment interaction, *p* < 0.01. WT, WT + sim, *Mstn^−/−^* and *Mstn^−/−^* + sim mice (WT, *n* = 6 mice; WT + simvastatin, *n* = 6 mice; *Mstn^−/−^*, *n* = 7 mice; *Mstn^−/−^* + simvastatin, *n* = 6 mice, *p < 0.05, **p < 0.01, ***p < 0.001). (I) Immunofluorescence staining of GLP‐1 in ileal tissues. WT, WT + sim, *Mstn^−/−^* and *Mstn^−/−^* + sim mice (WT, *n* = 5 mice; WT + simvastatin, *n* = 5 mice; *Mstn^−/−^*, *n* = 6 mice; Mstn^−/−^ + simvastatin, *n* = 6 mice, *p < 0.05, **p < 0.01, ***p < 0.001). (J) Immunofluorescence staining of SGLT1 in ileal tissues. WT, WT + sim, *Mstn^−/−^* and *Mstn^−/−^* + sim mice (WT, *n* = 5 mice; WT + simvastatin, *n* = 5 mice; *Mstn^−/−^*, *n* = 6 mice; Mstn^−/−^ + simvastatin, *n* = 6 mice). Data are presented as mean ± s.e.m. *p* values were determined by one‐way ANOVA followed by Tukey's post hoc test (B, E–I and the AUC analysis in F), two‐sided unpaired Student's t‐test (C), two‐way repeated‐measures ANOVA followed by Tukey's post hoc test (F). *p < 0.05 was considered statistically significant.

## 3 Discussion

Although impaired incretin action of pancreatic β cells is a major feature of type 2 diabetes, intestinal nutrient sensing and endogenous GLP‐1 secretion remain important components of postprandial blood glucose regulation [39, 40]. In this study, we identified the functional mechanism by which MSTN regulates intestinal SGLT1 under metabolic stress. MSTN is a well‐established negative regulator of skeletal muscle growth and energy metabolism [41, 42]. Here we show that MSTN is expressed in the intestine and that its deficiency enhances intestinal GLP‐1 synthesis and secretion, accompanied by improved insulin release and glucose tolerance under HFD conditions. This phenotype was consistently observed in both male and female mice, indicating that the regulation of intestinal GLP‐1 by MSTN is not a sex‐specific phenomenon. Notably, MSTN deficiency increased the relative pancreatic β‐cell area without markedly altering α‐cell distribution, suggesting that changes in β‐cell mass may also contribute to the enhanced insulin response. Previous studies have shown that GLP‐1 can promote β‐cells proliferation and survival [43, 44], providing one possible explanation for this observation, although a direct causal relationship was not examined in the present study. Importantly, blockade of GLP‐1R signaling abolished the improvement in glucose tolerance associated with intestinal Mstn deletion. Experiments using intestinal organoids and STC‐1 cells further localized this effect to intrinsic regulation within intestinal epithelial and enteroendocrine cells, rather than merely reflecting a secondary consequence of systemic metabolic remodeling. These findings extend the metabolic role of MSTN beyond skeletal muscle to the intestine and support a previously unrecognized role for MSTN as an upstream inhibitory signal of glucose‐dependent incretin output (Figure 8).

**FIGURE 8:**
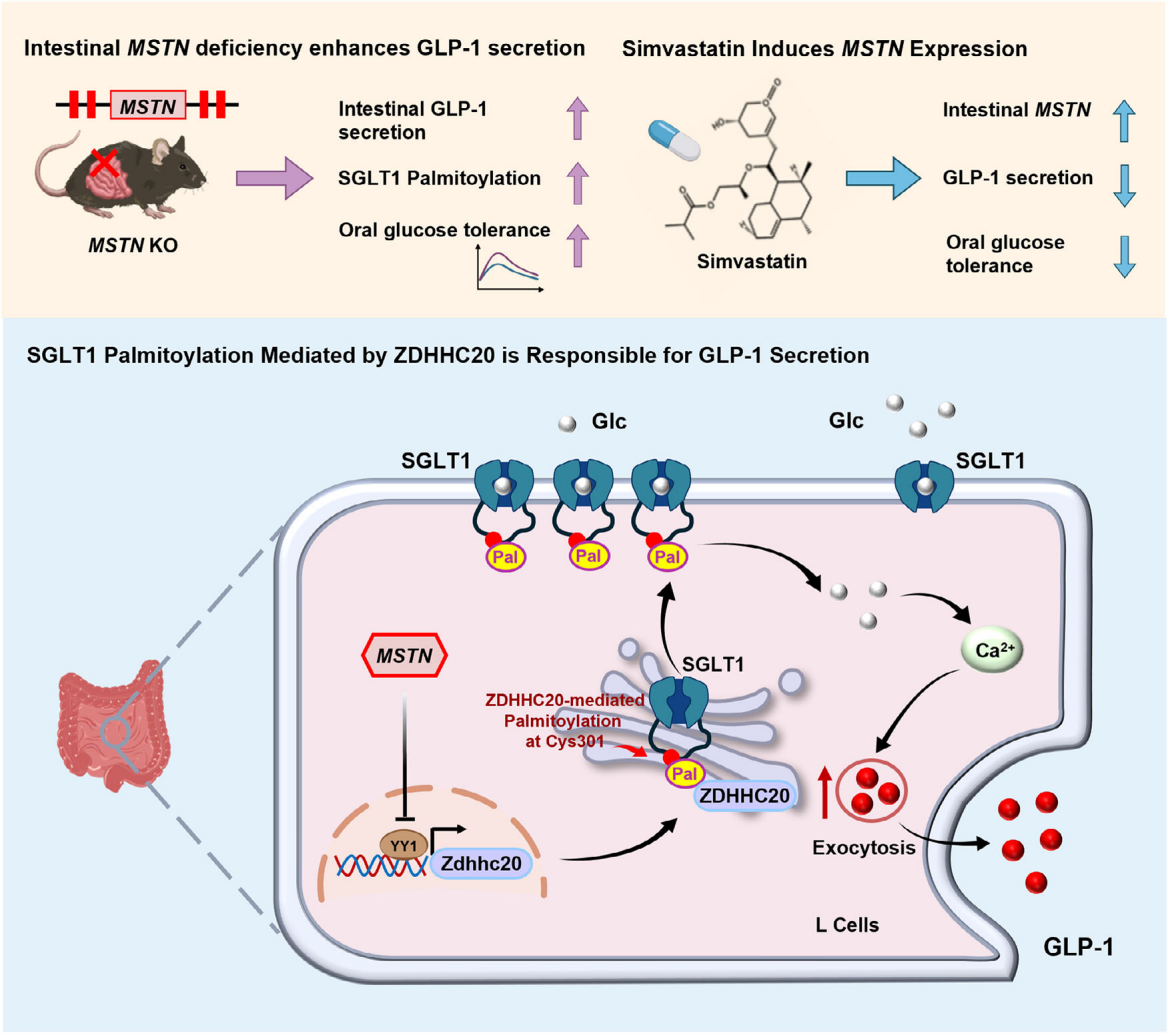
Intestinal MSTN deficiency enhances GLP‐1 secretion and glucose tolerance through ZDHHC20‐dependent SGLT1 S‐palmitoylation. Simvastatin increases intestinal MSTN expression and impairs GLP‐1 secretion. While intestinal MSTN deficiency promotes YY1‐associated ZDHHC20 expression, leading to increased SGLT1 S‐palmitoylation at Cys301 and enhanced plasma membrane localization in intestinal L cells. This mechanism amplifies glucose‐stimulated GLP‐1 secretion and improves glucose tolerance in HFD‐fed mice.

The function of intestinal SGLT1 is closely linked to its apical membrane localization [45]. Increased SGLT1 abundance at the brush border of small intestinal villi is directly associated with enhanced glucose absorptive capacity and promotes glucose‐stimulated GLP‐1 secretion [46]. Here, we demonstrate that MSTN deficiency markedly increases the accumulation of SGLT1 at the plasma membrane. This observation is consistent with the regulatory principles of other metabolism‐related membrane proteins, such as GLUT4 and CD36, for which membrane translocation and membrane residence often determine functional output more directly than total protein abundance [27, 47]. Although intestinal glucose absorption and transport primarily depend on SGLT1 and GLUT2 [26, 48, 49], GLUT2 was not markedly altered in either cytosolic or membrane fractions in our study. Importantly, phlorizin blockade abolished the MSTN inhibition‐induced increases in 2‐NBDG uptake and GLP‐1 secretion, further supporting SGLT1 as the key effector through which MSTN deficiency enhances intestinal glucose sensing and incretin output. Previous studies have also shown that reducing apical SGLT1 in intestinal epithelial cells decreases glucose absorption and transiently lowers postprandial glycemia [50], whereas HFD is associated with decreased SGLT1 expression, impaired glucose‐stimulated GLP‐1 release, and disruption of this negative feedback pathway [51]. Our findings echo these studies, suggesting that increasing the functional availability of membrane‐localized SGLT1 preferentially amplifies an intestinal glucose‐sensing‐GLP‐1 output pathway that favors glycemic control. SGLT1‐dependent glucose sensing is not restricted to GLP‐1‐producing L cells and also contributes to glucose‐stimulated GIP secretion from K cells [52, 53]. Notably, Mstn deficiency increased Gcg and Pcsk1 expression and enhanced GLP‐1 secretion, whereas Gip expression and circulating GIP levels were not detectably altered under the same conditions. Co‐staining and quantitative analysis demonstrated increased SGLT1 membrane enrichment in GLP‐1‐positive L cells in Mstn‐deficient mice. These findings suggest that MSTN deficiency enhances SGLT1‐dependent glucose responsiveness in the context of an increased GLP‐1 biosynthetic program. Nevertheless, the basis for the preferential enhancement of GLP‐1, rather than GIP, remains unclear. And the specific effects of K cells on SGLT1 and ZDHHC20 were not detected. Future studies assessing ZDHHC20 expression, SGLT1 palmitoylation, and plasma membrane localization specifically in K cells, together with dynamic measurements of GIP secretion, will be required to define the cell‐type‐specific effects of MSTN deficiency across enteroendocrine populations.

SGLT1 membrane trafficking has been reported to be regulated by rapid translocation mediated by kinases such as PKC and PKA, as well as by RS1/RSC1A1‐associated exocytotic processes [54, 55]. In recent years, palmitoylation has attracted increasing attention as a mechanism regulating the membrane localization of membrane proteins [29, 56]. However, the post‐translational mechanisms that control the functional availability of SGLT1 at the plasma membrane remain incompletely understood. Our findings identify SGLT1 S‐palmitoylation as a previously unrecognized mechanism controlling SGLT1 membrane enrichment and glucose‐responsive function. We proposed that SGLT1 palmitoylation promotes its plasma membrane localization and increases its functional availability at the cell surface, thereby facilitating GLP‐1 secretion. To test this hypothesis, we first directly demonstrated that SGLT1 undergoes palmitoylation using ABE and Alk‐C16 labeling assays. Second, we showed that inhibition of palmitoylation with 2‐BP attenuated GLP‐1 secretion. Finally, site‐directed mutagenesis and functional assays identified Cys301 as a critical functional palmitoylation site of SGLT1. Palmitoylation is a reversible process dynamically regulated by ZDHHC family palmitoyl acyltransferases and APT‐family depalmitoylating enzymes [57, 58]. ZDHHC family members transfer palmitoyl groups to cysteine residues of substrate proteins through their conserved DHHC catalytic domains, whereas depalmitoylating enzymes such as APT1/2 and ABHD17 remove this modification [59, 60]. We therefore asked whether a specific ZDHHC enzyme participates in SGLT1 regulation. By integrating intestinal expression profiles, subcellular localization features, and consistent changes observed across systemic *Mstn* deficiency, intestinal‐specific *Mstn* deficiency, and *Mstn*‐silenced cellular models, we systematically screened candidate ZDHHC family members and focused on ZDHHC20. Intriguingly, structural prediction suggested a potential interaction interface between ZDHHC20 and SGLT1, and Co‐IP analysis further supported their association within the same protein complex. Functionally, ZDHHC20 promoted SGLT1 palmitoylation, whereas HBAAV2/9‐mediated *Zdhhc20* overexpression in intestinal epithelial cells increased SGLT1 membrane accumulation and enhanced GLP‐1 release. Although the present study did not systematically evaluate the contribution of depalmitoylating enzymes such as APT1/2 and ABHD17, our current evidence supports a direct post‐translational mechanism in which ZDHHC20 enhances SGLT1 palmitoylation, thereby increasing its plasma membrane abundance and functional availability to regulate glucose‐stimulated GLP‐1 output.

YY1 is a multifunctional transcriptional regulator implicated in diverse biological processes, including cell proliferation, differentiation, and metabolic control [61]. To identify upstream regulators of *Zdhhc20*, we used online prediction tools to identify candidate transcription factors for *Zdhhc20* and integrated these results with our transcriptomic data, which ultimately highlighted the highly expressed transcription factor YY1. Unexpectedly, a prominent YY1 ChIP‐seq enrichment peak was observed in the *Zdhhc20* promoter region, and a putative YY1‐binding motif (CAAGTAGC) was also identified within the promoter sequence, providing a theoretical basis for the involvement of YY1 in *Zdhhc20* transcriptional regulation. Consistent with this inference, *Yy1* and *Zdhhc20* showed a significant positive correlation in public intestinal transcriptomic datasets, and both were coordinately upregulated in *Mstn^−/−^* mice, *Mstn^ΔIE^* mice, and *Mstn*‐silenced cellular models. Yy1 knockdown reduced ZDHHC20 expression, and, importantly, simultaneous Yy1 knockdown attenuated the increase in ZDHHC20 induced by Mstn silencing. These findings support a YY1‐dependent regulatory step linking MSTN deficiency to ZDHHC20 upregulation and subsequent SGLT1 palmitoylation. Nevertheless, whether YY1 directly binds to the *Zdhhc20* promoter and drives its transcriptional activation remains to be further validated.

Statins are among the most widely used agents for lowering lipid‐driven cardiovascular risk [62]. With the expanding use of statins, increasing attention has been paid to their metabolic adverse effects and muscle‐related side effects [7]. In addition to common muscle symptoms, statins can cause dose‐dependent elevations in blood glucose and increase the risk of new‐onset diabetes. Among them, lipophilic statins such as simvastatin are closely associated with myocyte toxicity, muscle atrophy‐like changes, and activation of skeletal muscle catabolic programs [35, 63]. Previous studies have shown that simvastatin upregulates skeletal muscle myostatin expression and promotes atrophy‐associated signaling [7]. Other evidence also suggests that statins may affect glucose metabolism by reducing circulating GLP‐1 levels and exacerbating insulin resistance [64]. The effects of statins on incretin secretion appear to may vary according to statin type, dose, duration of exposure, and host metabolic context [64, 65]. In our experimental model, simvastatin treatment increased intestinal MSTN expression and was accompanied by reduced GLP‐1 output and impaired glucose tolerance. Under simvastatin exposure, Mstn‐deficient mice maintained higher GLP‐1 output and better glucose tolerance than WT mice. These findings suggest that MSTN may participate in linking statin‐associated glucose metabolic dysfunction to impaired intestinal incretin homeostasis. A recent study reported that statins aggravate insulin resistance through a microbiota‐dependent reduction in circulating GLP‐1, involving altered gut microbiota and bile acid metabolism through the Clostridium sp.‐bile acid axis [64]. This mechanism and the MSTN‐ZDHHC20‐SGLT1 palmitoylation pathway identified in our study are not mutually exclusive. Rather, they may represent distinct and potentially complementary levels of intestinal incretin regulation during statin exposure. The microbiota–bile acid pathway emphasizes regulation of GLP‐1 secretion by luminal microbial and metabolic signals, whereas our findings identify a cell‐intrinsic mechanism in which MSTN influences SGLT1 membrane availability through ZDHHC20‐dependent palmitoylation. Whether MSTN signaling interacts with microbiota‐derived metabolites or bile acid signaling during statin‐associated glucose dysregulation remains an important question for future investigation.

It should be noted that the simvastatin intervention experiment was performed using global Mstn knockout mice. Although MSTN deficiency attenuates simvastatin‐induced impairment of GLP‐1 secretion and glucose tolerance, we did not perform the corresponding simvastatin intervention in intestinal epithelial‐specific Mstn knockout mice. Therefore, the present data cannot fully distinguish the contribution of intestinal MSTN deficiency from systemic effects of global MSTN deletion in this setting. Future studies using intestinal epithelial‐specific Mstn knockout mice under statin treatment and human‐relevant intestinal models will be important for defining the tissue‐specific role and translational relevance of the MSTN‐ZDHHC20‐SGLT1 palmitoylation pathway.

In summary, our study reveals a mechanism by which MSTN deficiency regulates SGLT1 S‐palmitoylation as a previously unrecognized post‐translational mechanism controlling its plasma membrane availability and glucose‐responsive function. This modification is that ZDHHC20 promotes SGLT1 palmitoylation at Cys301, resulting in increased plasma membrane availability of SGLT1 and thereby promoting glucose‐stimulated GLP‐1 secretion. This mechanism may represent a regulatory node connecting intestinal nutrient sensing, gut‐derived GLP‐1 secretion, and glucose metabolic homeostasis. MSTN‐targeting strategies already have a degree of translational precedent, as MSTN inhibitors such as MYO‐029 have entered clinical studies for muscle‐wasting disorders. In addition, ZDHHC20, as a key palmitoyl acyltransferase linking MSTN signaling to SGLT1 membrane localization, provides a new entry point for improving incretin responses through the regulation of post‐translational modification of specific membrane proteins.

## 4 Methods

### 4.1 Animals and Diets

Specific pathogen‐free (SPF) global Mstn‐knockout mice (*Mstn^−/−^*) were obtained from Shanghai Model Organisms Center (Shanghai, China). *Mstn^fl/fl^* mice were crossed with Villin‐Cre transgenic mice to generate intestinal epithelial cell‐specific Mstn‐knockout mice (*Mstn^ΔIE^*), which were provided by Cyagen Biosciences (Suzhou, China) and were generated by crossing mice carrying floxed *Mstn* alleles with Villin‐Cre transgenic mice. The primer sequences for Genotyping are listed in Table S1. All lines were maintained on a C57BL/6J background.

Mice were housed in an SPF facility at 20°C–24°C and 40%–60% humidity under a 12 h light/12 h dark cycle, with ad libitum access to food and water. Standard chow diet was obtained from Huafukang Bioscience (1032, Beijing, China), and high‐fat diet (HFD, 60% kcal from fat) from Xietong Pharmaceutical Bioengineering (XTHF60, Jiangsu, China).

Experiments were performed in male mice unless otherwise specified in the corresponding figure legends. Experiments under standard chow conditions were performed using 8–12 weeks old mice. For the HFD model, mice were started on HFD at 8 weeks of age and maintained on HFD for 12 weeks before metabolic testing and sample collection. For simvastatin treatment experiments, 8‐week‐old mice were administered simvastatin (S6196, Sigma) by oral gavage at 40 mg/kg/daily for 4 weeks. For intestinal adeno‐associated virus (AAV)‐mediated overexpression experiments, C57BL/6J mice were started on HFD at 8 weeks of age. After 4 weeks of HFD feeding, mice received AAV injection and were then maintained on HFD for an additional 4 weeks before sample collection and analysis.

Sample size was not determined by formal power calculation but was guided by prior studies and pilot experiments. All animal procedures were approved by the Animal Ethics Committee of Yanbian University (approval no. YDSY20220120004) and conducted in accordance with relevant institutional guidelines.

### 4.2 Metabolic Studies

For indirect calorimetry, mice were placed singly in metabolic cages (Columbus Instruments, USA) with ad libitum access to food and water. Following a 24 h acclimation period, metabolic parameters were recorded continuously for 72 h, including food intake, VO_2_, VCO_2_, respiratory exchange ratio (RER), and heat production as an index of energy expenditure. Data acquisition and calculations were performed using Oxymax software.

### 4.3 Adeno‐Associated Virus Transduction

The intestinal *Zdhhc20* overexpression model was established based on a previously reported method with appropriate modifications [31]. Briefly, SPF C57BL/6J mice were anesthetized, and the abdominal cavity was opened to expose the distal ileum. A segment near the ileocecal junction was selected, and vascular clamps were placed at both ends to temporarily block the intestinal contents and prevent diffusion of the viral solution. A longitudinal incision of approximately 3 mm was then made in the intestinal wall, and the intestinal lumen was gently flushed with sterile saline using an insulin syringe. A total of 200 µL AAV solution was then injected into the intestinal lumen through the incision. The AAV vectors used in this study were HBAAV2/9‐mCherry control virus and HBAAV2/9‐m‐Zdhhc20‐HA‐mCherry overexpression virus (HanBio, Shanghai, China), both at a titre of 1.3 × 10^12^ vg/mL. After intraluminal delivery, the viral solution was retained in the ileal segment for 20 min. The vascular clamps were then removed, and the intestinal lumen was gently flushed with 200 µL sterile saline. The intestinal incision was closed with 10‐0 sutures, followed by layered closure of the abdominal wall. Mice were allowed to recover after surgery, and tissues and functional data were collected 4 weeks after viral injection.

### 4.4 Oral Glucose Tolerance Test

For oral glucose tolerance tests (OGTTs), mice were fasted for 16 h before testing. Glucose (PHR1000, Sigma–Aldrich) was dissolved in sterile saline and delivered by oral gavage at 2 g/kg body weight. Blood glucose was measured from tail‐vein blood at 0 min before gavage and at 15, 30, 60, 90, and 120 min thereafter. Glucose tolerance curves were plotted, and the area under the curve (AUC) was calculated. For GLP‐1 receptor blockade, mice received an intraperitoneal injection of the GLP‐1R antagonist Exendin(9‐39) (Ex 9–39, HY‐P0264, MedChemExpress) 1 h before glucose gavage. Exendin (9‐39) was dissolved in DMSO and diluted in sterile saline before administration at 50 µg per mouse in a final volume of 200 µL.

### 4.5 ELISA

After 12 weeks of chow or HFD feeding, mice were fasted for 16 h according to the experimental design. Blood samples were collected before glucose gavage (0 min) and 15 min after glucose gavage. To prevent degradation of active GLP‐1, blood collection tubes were preloaded with DPP‐4 inhibitor (DPP4‐M, Sigma–Aldrich). Blood samples were centrifuged at 4°C to isolate serum, which was stored until analysis. Active GLP‐1 (EGLP‐35K, Sigma–Aldrich), GIP (MM‐0409M1, MEIMIAN), and insulin (CSB‐E05071m, Cusabio) concentrations were measured using ELISA kits according to the manufacturers’ instructions.

### 4.6 Intestinal Organoid Culture

Small intestinal organoids were established based on previously reported methods with appropriate modifications [66]. Organoids were derived from ileal tissues of WT and *Mstn^−/−^* mice after 12 weeks of HFD feeding. After euthanasia, approximately 6 cm of ileal tissue was collected, flushed to remove intestinal contents, opened longitudinally, and gently scraped to remove mucus and villi. Tissues were washed with prechilled PBS (AM9625, Thermo Fisher Scientific) containing 1% penicillin–streptomycin (15140122, Gibco) and then cut into approximately 2 × 2 mm pieces. Tissue pieces were incubated in PBS containing 5 mM EDTA (E1170, Solarbio) at 4°C for 30 min, followed by mechanical shaking to release intestinal crypts. The suspension was washed with 0.1% BSA, filtered through a 70 µm cell strainer, and centrifuged to collect crypts. Crypts were resuspended at approximately 200 crypts per 10 µL Matrigel (0827035, Mogengel) and seeded into 24‐well culture plates (3524, Costar). After Matrigel polymerization, organoid culture medium (MA‐0817H006LP, Mogengel) was added, and organoids were cultured at 37°C in a 5% CO2 incubator. Organoids were used for subsequent experiments or immunofluorescence staining after 5 d of culture.

### 4.7 Cell Culture

STC‐1 mouse enteroendocrine cells (CRL‐3254) and HEK293T cells (CRL‐11268) were obtained from ATCC. Cells were routinely screened for mycoplasma contamination and maintained in DMEM containing 10% fetal bovine serum (F0850, Sigma–Aldrich) and 1% penicillin–streptomycin. Cultures were kept at 37°C in a humidified atmosphere with 5% CO_2_.

### 4.8 GLP‐1 Secretion Assay

Organoids cultured for 5 d or STC‐1 cells subjected to the indicated treatments were used for GLP‐1 secretion assays. Before the assay, samples were preincubated in balanced salt buffer containing 20 nM DPP‐4 inhibitor. The buffer contained 1.2 mM CaCl_2,_ 1.2 mM MgCl_2_, 4.5 mM KCl, 140 mM NaCl, and 20 mM HEPES. Samples were then incubated for 2 h under the following conditions: (1) basal buffer; (2) glucose stimulation buffer containing 10 mM glucose; or (3) forskolin/IBMX stimulation buffer containing 10 mM glucose, 10 µM forskolin, and 10 µM IBMX. For 2‐bromopalmitate (2‐BP) treatment experiments, cells were pretreated with 2‐BP for 6 h before the GLP‐1 secretion assay. For phlorizin inhibition experiments, cells were incubated in the presence or absence of phlorizin (HY‐N0143, MedChemExpress) for 2 h. Supernatants were subsequently collected for active GLP‐1 quantification by ELISA according to the manufacturer's protocol. Percentage of GLP‐1 secretion was calculated by measuring GLP‐1 levels in the supernatants and cell lysates and normalized to WT organoids in parallel on the same day [67, 68].

### 4.9 Plasmid Construction and Cell Transfection

Expression plasmids encoding wild‐type SGLT1, site‐directed SGLT1 mutants, and ZDHHC20 were generated by GenePharma (Suzhou, China). The SGLT1 overexpression constructs, including wild‐type SGLT1 and the C301S and C560S mutants, contained a C‐terminal GFP tag and a Flag tag for visualization and immunoblot detection. siRNA sequences targeting Mstn, Zdhhc20 and Yy1 are provided in Table S2. Transfections were performed with Lipofectamine 3000 reagent (L3000015, Thermo Fisher Scientific) following the manufacturer's protocol. The concentrations of plasmids and siRNAs were optimized and applied according to the manufacturers’ recommendations. Cells were collected at the indicated time points after transfection for subsequent RNA, protein, and functional analyses.

### 4.10 Co‐Immunoprecipitation

Cells were lysed in NP‐40 lysis buffer (HY‐K1002, MedChemExpress) containing protease and phosphatase inhibitors. After centrifugation to remove insoluble material, protein concentrations were measured by BCA assay. For each immunoprecipitation, 1 mg of total protein lysate was incubated with 2 µg anti‐HA or anti‐Flag antibody overnight at 4°C with gentle rotation. Protein A/G magnetic beads (88802, Thermo Fisher Scientific) were washed in advance, added to the antibody–lysate mixture and incubated for 1 h at 4°C. The bead‐bound immune complexes were collected using a magnetic separator, washed three times with lysis buffer and eluted in SDS sample buffer. Eluted proteins were then analysed by immunoblotting.

### 4.11 Fluorescence In Situ Hybridization

*Mstn* mRNA was detected in tissue sections using an RNA fluorescence in situ hybridization (FISH) kit (F21401, GenePharma). Cy3‐labelled probes targeting MSTN, together with corresponding negative‐control probes, were designed and synthesized by GenePharma (Suzhou, China). The probe sequences were as follows: MSTN probe, 5′‐GCAGCAATCAGCATGAACAGCTCATTTAGATCCACTGGGCATGGCTTCTATTCTGGAGTA‐3′; negative control probe, 5′‐TGCTTTGCACGGTAACGCCTGTTTT‐3′. Paraffin sections were baked at 60°C and then subjected to deparaffinizati‐on, gradient ethanol rehydration, and proteinase K permeabilization. Prehybridization was performed according to the manufacturer's instructions, followed by overnight hybridization with Cy3‐labeled probes at 37°C in the dark. After hybridization, sections were stringently washed with SSC buffer to reduce nonspecific background signal. Nuclei were counterstained with DAPI, and sections were mounted with antifade mounting medium. Images were acquired and analyzed using a confocal laser scanning microscope (FV3000, Olympus, Tokyo, Japan).

### 4.12 Plasma Membrane Protein Isolation

Plasma membrane and cytosolic protein fractions were prepared from cells and tissues using a plasma membrane/cytosol membrane protein extraction kit (ab65400, Abcam) following the manufacturer's protocol. In brief, samples were rinsed twice with ice‐cold PBS and lysed in homogenization buffer on ice. The lysates were mechanically disrupted with 30–50 strokes using a Dounce homogenizer and cleared by centrifugation at 700 × *g* for 5 min to remove nuclei and intact cells. The resulting supernatant was centrifuged at 10 000 × *g* for 30 min at 4°C to separate the crude membrane‐enriched fraction from the cytosolic fraction.

For plasma membrane purification, the membrane‐enriched pellet was resuspended in upper‐phase solution, combined with lower‐phase solution and incubated on ice for 5 min. After centrifugation at 1000 × g for 5 min, the upper phase was collected and further centrifuged at 15 000 × g for 30 min. The final plasma membrane pellet was resuspended in extraction buffer and subjected to immunoblot analysis.

### 4.13 Acyl‐Biotin Exchange Assay

Protein S‐palmitoylation was detected using the acyl‐biotin exchange (ABE) assay [69]. Cells were lysed in lysis buffer containing 25 mM N‐ethylmaleimide (NEM, 23030, Thermo Fisher Scientific), 50 mM Tris‐HCl, 5 mM EDTA, 150 mM NaCl, and 4% SDS, pH 7.4, supplemented with protease inhibitors. Samples were incubated with agitation at room temperature for 2 h to block free thiol groups. Proteins were then precipitated using chloroform/methanol/water (1:4:3, v/v/v) to remove residual NEM. After resolubilization, protein samples were divided equally into two aliquots and treated with either 1 M hydroxylamine (HA, 438227, Sigma–Aldrich) or 1 M NaCl as a negative control for 3 h at room temperature to cleave thioester bonds and expose thiol groups at palmitoylation sites. Samples were then labeled with 5 mM Biotin‐HPDP (21341, Thermo Fisher Scientific). After a second precipitation step, proteins were resuspended in resuspension buffer containing 50 mM Tris‐HCl, 2% SDS, 8 M urea, and 5 mM EDTA, pH 7.4. A portion of each sample was retained as input control. The remaining samples were diluted with PBS and incubated with streptavidin magnetic beads (HY‐K0208, MedChemExpress) overnight at 4°C. Beads were washed with PBS containing 0.2% SDS, and bound proteins were eluted with SDS loading buffer and analyzed by immunoblotting.

### 4.14 Click Chemistry Assay

Cells were transfected with wild‐type or mutant SGLT1 plasmids for 48 h, followed by incubation with the alkynyl palmitic acid analog Alk‐C16 (100 µM) for an additional 12 h to label S‐palmitoylated proteins. Cells were washed with PBS and lysed in lysis buffer containing 50 mM Tris‐HCl, 150 mM NaCl, and 4% SDS, pH 7.4. Samples were sonicated to fully solubilize proteins and shear nucleic acids. Lysates were clarified by centrifugation to remove insoluble material, and protein concentrations were determined. Click chemistry was carried out in the dark at 37°C for 2 h using 50 µM biotin‐azide, 1 mM CuSO_4_, 1 mM TCEP and 100 µM TBTA. Proteins were then precipitated with chloroform/methanol/water (1:4:3, v/v/v), resuspended in 2% SDS‐containing buffer and diluted with PBS. Biotin‐labeled proteins were enriched with streptavidin magnetic beads for 1 h at 4°C. After extensive washing, bead‐bound proteins were eluted by boiling in SDS loading buffer and analysed by western blotting.

### 4.15 Immunohistochemistry and Immunofluorescence

Mouse ileal tissues were collected, flushed thoroughly with PBS to remove intestinal contents, fixed in 4% paraformaldehyde (PFA), paraffin‐embedded, and sectioned at 5 µm. Sections were subjected to deparaffinization, rehydration, and antigen retrieval before immunohistochemistry (IHC) or immunofluorescence (IF) staining. Small intestinal organoids and STC‐1 cells were fixed with 4% PFA before further processing. All samples were permeabilized and blocked before antibody incubation. Organoids were blocked at room temperature for 2 h in PBS containing 10% goat serum and 0.3% Triton X‐100. For immunofluorescence staining, tissue sections and organoids were mainly used to detect GLP‐1, SGLT1, GLUT2, Occludin, and ZO‐1, whereas STC‐1 cells were used for YY1 nuclear localization analysis. For staining of three‐dimensional organoids, primary antibodies were diluted in buffer containing 5% goat serum and 0.1% Triton X‐100 and incubated at 4°C for 48 h to improve antibody penetration. MSTN was detected by immunohistochemistry using the DAB method. After primary antibody labelling, specimens were rinsed with PBS and exposed to fluorescence‐conjugated secondary antibodies. DAPI was used for nuclear counterstaining, and images were captured with a confocal laser scanning microscope (Olympus).

### 4.16 2‐NBDG Glucose Uptake Assay

At 48 h after transfection, cells were washed three times with PBS and incubated in serum‐free medium containing 100 µM 2‐NBDG (HY‐116215, MedChemExpress) at 37°C for 1 h to assess glucose uptake. The staining solution was then removed, and cells were thoroughly washed with PBS. Images were acquired and analyzed using a fluorescence microscope (Olympus).

For 2‐bromopalmitate (2‐BP) treatment experiments, cells were pretreated with 50 µM 2‐BP for 6 h before the assay. For phlorizin treatment experiments, cells were pretreated with 100 µM phlorizin for 30 min before the assay.

### 4.17 Western Blotting

Proteins were extracted from tissues or cultured cells using RIPA lysis buffer (P0038, Beyotime) containing protease and phosphatase inhibitors. Protein concentration was measured with a BCA assay kit (P0012, Beyotime), and 20 µg of total protein per sample was resolved by SDS–PAGE before transfer to PVDF membranes (MilliporeSigma). After blocking in 5% non‐fat milk for 1 h at room temperature, membranes were probed with primary antibodies at 4°C overnight, followed by HRP‐conjugated secondary antibodies. Chemiluminescent signals were visualized using a GelView 5000 Plus Imaging System (BioLight, Guangzhou, China), and band densitometry was performed with ImageJ. Antibody details are listed in Table S3.

### 4.18 RNA Isolation and RT‐qPCR

Total RNA was isolated from cells or tissues with TRIzol reagent (LS1040, Promega). cDNA was generated using a reverse transcription kit (4897030001, Roche) following the manufacturer's protocol. Quantitative PCR was carried out on an Mx3005P system (Agilent, Santa Clara, CA, USA), and relative transcript abundance was calculated using the 2^−ΔΔCt^ method after normalization to the indicated housekeeping gene. Primers were synthesized by Sangon Biotech (Shanghai, China), and the sequences are provided in Table S4.

### 4.19 Transcriptomics Sequencing

Total RNA was extracted from ileal tissue, and library construction and transcriptome sequencing were performed by Novogene. Sequencing was carried out on the Illumina platform (NEB, USA). Raw sequencing data were processed using fastp. A reference genome index was constructed using HISAT2 (v2.2.1), and paired‐end reads were aligned to the reference genome. Gene expression levels were quantified using featureCounts (v2.0.6). Differential expression analysis was performed using the DESeq2 R package (v1.42.0). This dataset has been uploaded to the SRA database (sample number: SAMN63240443, SAMN63240444, SAMN63240445, SAMN63240446, SAMN64340447, SAMN62340448, SAMN663240449, SAMN363240450, dataset number: PRJNA1531303).

### 4.20 Statistical Analysis

Data are shown as mean ± s.e.m. Statistical analyses and graph generation were performed in GraphPad Prism 10.1.2. Data distribution was evaluated using the Shapiro–Wilk test before group comparisons. For normally distributed datasets, two‐group comparisons were analysed using two‐sided unpaired Student's t‐tests, whereas comparisons involving more than two groups were performed by one‐way or two‐way ANOVA with Tukey's multiple‐comparison test. For datasets that did not meet normality assumptions, two‐sided Mann–Whitney tests or Kruskal–Wallis tests followed by Dunn's post hoc test were used, as appropriate. Time‐course data were analysed by two‐way repeated‐measures ANOVA. P values <0.05 were considered statistically significant. Statistical significance was defined as *p < 0.05, **p < 0.01, and ***p < 0.001.

## Author Contributions

J.D.K. and L.H.Q. supervised and conceived the study. S.Z.H. performed the majority of the experiments, analyzed the data, prepared the figures, and participated in study design. S.Y.C. and K.S. designed and performed the cell biology experiments and participated in the collection and analysis of clinical samples. Z.B.L. contributed to experimental design, provided scientific guidance on methodology, and drafted the initial version of the manuscript. S.Y.C., K.S., Y.Q.L., B.H.Q., Z.Y.L., X.Y.L., S.T.L., M.F.X., X.Y.X., X.F. H.C. and R.H.Z. assisted with sample collection and animal experiments. Y.Q.L., B.H.Q. and H.C. performed molecular docking and protein structure analyses. Z.Y.L., X.Y.L., S.T.L. and M.F.X. assisted with data analysis. X.J.Y. contributed to study design and provided guidance on data interpretation and manuscript preparation. J.D.K., X.J.Y. and L.H.Q. secured funding and supervised the project. All authors reviewed, edited, and approved the final manuscript.

## Funding

This work was supported by the National Natural Science Foundation of China 32372971, 32260817 (to J.D.K), and 32260026 (to L.H.Q). The Changbai Mountain Talent Project of Jilin Province 000007 (to L.H.Q).

## Ethics Statement

All animal procedures were approved by the Animal Ethics Committee of Yanbian University (approval no. YDSY20220120004) and conducted in accordance with relevant institutional guidelines.

## Conflicts of Interest

The authors declare no conflicts of interest.

## Supporting information




**Supporting File**: advs78302‐sup‐0001‐SuppMat.docx.

## Data Availability

The data that support the findings of this study are available from the corresponding author upon reasonable request.
