# Supplementary material for: Loss of Intestinal MSTN Promotes GLP‐1 Secretion Through SGLT1 Palmitoylation
Source: Adv Sci (Weinh). 2026 Oct 14:e78302. Online ahead of print. doi: 10.1002/advs.78302 (PMC13669553; doi:10.1002/advs.78302)
Supplement: Supplementary file 1 [file ADVS-9999-e78302-s001.docx]

Supporting Information

Loss of intestinal MSTN promotes GLP-1 secretion through SGLT1 palmitoylation

Sheng-Zhong Han^1^, Shuang-Yan Chang ^1^, Zhaobo Luo^3^, Ke Sun^1^, Biaohu Quan^1, 4^, Yanqiu Lv^5^, Zhouyan Li^1^, Xinyue Liu^1^, Sitong Lv^1^, Meifu Xuan^1, 4^, Xiaoyufan Xu^1^, Xue Fang^1^, Hang Chang^1^, Rihuan Zhang^1^, Xi-Jun Yin^1, 4*^, Lin-Hu Quan ^2*^, Jin-Dan Kang^1, 4*^

**Figure S1. MSTN knockout improves the intestinal barrier in HFD-fed mice and glucose intolerance in female mice.** **A**, Immunoblot analysis of MSTN expression in the small intestine of chow diet-fed and HFD-fed mice (*n* = 5). **B**, Validation of *Mstn* knockout by agarose gel electrophoresis. **C**, Fluorescence in situ hybridization (FISH) of *Mstn* mRNA in skeletal muscle. Scale bar, 50 μm (*n* = 5). **D**, Immunoblot analysis of MSTN, GCG and PC1/3 expression in the small intestine of WT and *Mstn^-/-^* mice (n = 5, ****p* < 0.001). **E**, Serum GIP levels before (0 min) and 15 min after oral glucose administration (*n* = 5). **F**, Cumulative body weight gain analysis of WT and *Mstn^-/-^* mice (*n* = 5, ***p* < 0.01, ****p* < 0.001). **G**, Immunoblot analysis of tight junction proteins ZO-1 and Occludin in the small intestine (*n* = 5, **p* < 0.05, ****p* < 0.001). **H**, Representative images of tight junction proteins immuno-fluorescence staining in small intestine sections. Scale bar, 50 μm (*n* = 5). **I**, **J**, Serum active GLP-1 (**I**) and insulin (**J**) levels before (0 min) and 15 min after oral glucose administration in female WT and *Mstn^-/-^* mice (*n* = 5, **p* < 0.05, ***p* < 0.01, ****p* < 0.001). **K**, Co-immunofluorescence staining of glucagon and insulin in the pancreatic sections of female mice. Scale bar, 50 μm (*n* = 5, ***p* < 0.01). **L**, Blood glucose curves during OGTTs and corresponding AUC in female mice (n = 5, ***p* < 0.01). Data are presented as mean ± s.e.m. *p* values were determined by two-sided unpaired Student’s t-test (**D**, **E**, the Occludin analysis in **G**, **I**-**K**, and the AUC analysis in **L**), two-way repeated-measures ANOVA followed by Tukey’s post hoc test (**F, L**), or two-sided Mann-Whitney test (the ZO-1 analysis in **G**). **p* < 0.05 was considered statistically significant.

**Figure S2. MSTN deficiency improves glucose tolerance and enhances intestinal barrier-associated protein expression. A**, Validation of conditional MSTN knockout by agarose gel electrophoresis. **B,** Immunoblot analysis of MSTN, GCG and protein expression in intestinal tissues from *Mstn^fl/fl^* and *Mstn^ΔIE^* mice (n = 5, ***p* < 0.01, ****p* < 0.001). **C,** Immunoblot analysis of MSTN protein expression in muscle, heart, kidney, white adipose tissue, brown adipose tissue (BAT), and lung tissues from *Mstn^fl/fl^* and *Mstn^ΔIE^* mice (n = 5). **D,** Fasting blood glucose levels (n = 5, ***p* < 0.01). **E**, Quantification of islet α-cell area based on immunofluorescence staining of glucagon in pancreatic sections (*n* = 5). **F**, **G**, Carbon dioxide production (VCO_2_) (**F**), and Oxygen consumption (VO_2_) (**G**) analysis (*n* = 4). **H**, **I**, Blood glucose curves during OGTTs and corresponding AUC (**H**), Cumulative body weight gain (**I**) analysis of *Mstn^fl/fl^* and *Mstn^ΔIE^* mice fed with chow diet (*n* = 6). **J**, Relative mRNA expression of *Pcsk1*, *Gcg*, *Gip* and *Dpp4* in the small intestine (*n* = 6, **p* < 0.05, ***p* < 0.01). **K**, **L**, Serum active GLP-1 (**K**) and insulin (**L**) levels before (0 min) and 15 min after oral glucose administration in chow diet-fed *Mstn^fl/fl^* and *Mstn^ΔIE^* mice (*n* = 6, **p* < 0.05, ****p* < 0.001). Data are presented as mean ± s.e.m. *p* values were determined by two-sided unpaired Student’s t-test (**B, D-G**, the AUC analysis in **H,** and **J–L**) or two-way repeated-measures ANOVA followed by Tukey’s post hoc test (**H**, **I**). **p* < 0.05 was considered statistically significant.

**Figure S3. MSTN deficiency or knockdown promotes plasma membrane localization of SGLT1. A**, Immunofluorescence staining of SGLT1 and GLUT2 in small intestine sections from *Mstn^fl/fl^*, *Mstn^ΔIE^*, WT and *Mstn^-/-^* mice. Scale bar, 50 μm. **B**, Immunoblot analysis of SGLT1 and GLUT2 in plasma membrane and cytosolic fractions isolated from small intestine tissues of WT and *Mstn^-/-^* mice (*n* = 5, ***p* < 0.01). **C**, Immunoblot analysis of GCG and PC1/3 protein levels in STC-1 cells (*n* = 6, ***p* < 0.01, ****p* < 0.001). **D**, Relative mRNA expression of *Pcsk1*, *Gcg*, *Gip* and *Dpp4* in STC-1 cells (*n* = 6, **p* < 0.05). Data are presented as mean ± s.e.m. *p* values were determined by two-sided unpaired Student’s t-test (**B**-**D**) or Welch’s t-test (the *Mstn* analysis in **D**). **p* < 0.05 was considered statistically significant.

**Figure S4. Palmitoylation of SGLT1 induces GLP-1 secretion. A**, Immunoblot analysis of GCG and PC1/3 protein levels in STC-1 cells transfected with siNC or siMstn following 2-BP (50 μM) treatment (*n* = 3, **p* < 0.05, ***p* < 0.01, ****p* < 0.001). **B**, Sequence conservation analysis of the Cys301 and Cys560 sites of SGLT1 protein across different species. **C**, Immunoblot analysis of GCG and PC1/3 protein levels in STC-1 cells overexpressing WT SGLT1, C301S, or C560S mutants (*n* = 3, **p* < 0.05, ***p* < 0.01, ****p* < 0.001). **D**, 2-NBDG uptake levels in STC-1 cells overexpressing WT SGLT1, C301S, or C560S mutants. Scale bar, 50 μm (*n* = 3). Data are presented as mean ± s.e.m. *p* values were determined by one-way ANOVA followed by Tukey’s post hoc test (**A**, **C**). **p* < 0.05 was considered statistically significant.

**Figure S5. ZDHHC20 mediates SGLT1 S-palmitoylation. A**, Expression profiles and subcellular localization of different *Zdhhc* family members in the intestine. **B**, Relative mRNA expression of different *Zdhhc* isoforms in STC-1 cells transfected with siNC or siMstn (*n* = 6, **p* < 0.05, ***p* < 0.01, ****p* < 0.001). **C**, **D**, Immunoblot analysis of GCG and PC1/3 protein levels in STC-1 cell lysates under the indicated treatment conditions (*n* = 3, **p* < 0.05, ***p* < 0.01, ****p* < 0.001). **E**, **F**, Relative mRNA expression of *Zdhhc20*, *Gcg*, *Pcsk1*, and *Slc5a1* in STC-1 cells transfected with siZdhhc20 (**E**) or oeZdhhc20 (**F**) (*n* = 3, **p* < 0.05, ****p* < 0.001). **G**, 2-NBDG uptake levels in STC-1 cells following oeZdhhc20 transfection following 2-BP (50 μM) treatment. Scale bar, 50 μm (*n* = 3). Data are presented as mean ± s.e.m. *p* values were determined by two-sided unpaired Student’s t-test (**B**, **E** and **F**), one-way ANOVA followed by Tukey’s post hoc test (**C**, **D**), or Welch’s t-test (the Zdhhc18 analysis in **B**). **p* < 0.05 was considered statistically significant.

**Figure S6. AAV-mediated overexpression of Zdhhc20 in the intestine. A**, Mice injected with AAV-Control or AAV-Zdhhc20^oe^ were fed a HFD for 4 weeks, mCherry fluorescence signals in ileal sections (*n* = 6). **B**, Relative mRNA expression of *Zdhhc20* in the ileum (***p* < 0.01). **C**, Immunoblot analysis of ZDHHC20, GCG, and PC1/3 protein levels in ileal lysates (**p* < 0.05, ***p* < 0.01, ****p* < 0.001). Data are presented as mean ± s.e.m. *p* values were determined by Welch’s t-test (**B**) or two-sided unpaired Student’s t-test (**C**). **p* < 0.05 was considered statistically significant.

**Figure S7. YY1 mediates MSTN inhibition-induced ZDHHC20 transcription. A**, Immunoblot analysis of YY1 and ZDHHC20 protein levels in ileal tissues (*n* = 5, ***p* < 0.01). **B**, Relative *Yy1* mRNA expression in WT and *Mstn^-/-^* mice (*n* = 5, ***p* < 0.01). **C**, Relative *Yy1* mRNA expression in STC-1 cells (*n* = 5, **p* < 0.05, ****p* < 0.001). **D**, Immunofluorescence staining of YY1 in STC-1 cells transfected with siNC or siYy1. Scale bar, 50 μm (*n* = 6). **E**-**H**, Spearman correlation analysis of *Yy1* and *Zdhhc20* expression with *Gcg* and *Pcsk1* expression in the GSE240206 (**E**, **F**) and GSE288466 (**G**, **H**) datasets. Data are presented as mean ± s.e.m. *p* values were determined by two-sided unpaired Student’s t-test (**A**-**C**) or two-sided Spearman’s rank correlation (**E–H**). **p* < 0.05 was considered statistically significant.

**Table S1.** **The primer sequences for Genotyping**

| Genotype | Primer | Forward Primer (5′-3′) | Reverse Primer (5′-3′) |
| --- | --- | --- | --- |
| MSTN^-/-^ | Primer1 | CAGCGAGATTCATTGTG | CCAGTAGGAGCCATTCAG |
|  | Primer2 | AGCTTGCCCTCGACTGTAAC | AGGACAGCAGTCAGCAGAAC |
| MSTN^ΔIE^ | Primer1 | TGTAACTGCTCAGATTCCCAAAGA | GATTCTACTCCCCTTCCAGAGAAC |
|  | Primer2 | CCATAGGAAGCCAGTTTCCCTTC | TTCCAGGTATGCTCAGAAAACGC |

**Table S2. List of the siRNA sequences that were used**

| Target gene | Forward Primer (5′-3′) | Reverse Primer (5′-3′) |
| --- | --- | --- |
| Yy1 | CUCCUGAUUAUUCUGAAUATT | UAUUCAGAAUAAUCAGGAGTT |
| Mstn | GCCCAGUGGAUCUAAAUGATT | UCAUUUAGAUCCACUGGGCTT |
| Zdhhc20 | CUGUGCGUGAGUACUAUUUTT | AAAUAGUACUCACGCACAGTT |
| Negative Control | UUCUCCGAACGUGUCACGUTT | ACGUGACACGUUCGGAGAATT |

**Table S3. List of the antibody that were used**

| Antibody | Application | Source | Lot # | Dilution |
| --- | --- | --- | --- | --- |
| MSTN | IHC-P/WB | Santa | SC-134345 | 1:50/1:1000 dilution |
| Insulin | IHC-P | Abcam | ab181547 | 1:200/1:2000 dilution |
| GLP-1 | IHC-P | Bioss | BSM-0933M | 1:100/1:2000 dilution |
| GCG | WB | Proteintech | 15954-1-AP | 1:2000 dilution |
| Glucagon | IHC-P | Abcam | EP3070 | 1:100 dilution |
| ZO-1 | IHC-P/WB | Abcam | BS-34023R | 1:100/1:2000 dilution |
| Occludin | IHC-P/WB | Bioss | BS-10011R | 1:100/1:2000 dilution |
| β-actin | WB | Sigma-Aldrich | A1978 | 1:2000 dilution |
| SGLT1 | IHC-P/WB | Proteintech | 30861-1-AP | 1:100/1:2000 dilution |
| GLUT2 | IHC-P/WB | Proteintech | 20436-1-AP | 1:100/1:2000 dilution |
| HA | WB | Abway | M20003 | 1:5000 dilution |
| Flag | WB | Sigma-Aldrich | MAB3118 | 1:5000 dilution |
| ATP1A1 | WB | Abcam | ab76020 | 1:2000 dilution |
| ZDHHC20 | WB | Bioss | bs-7418R | 1:2000 dilution |
| PC1/3 | WB | Abcam | ab220363 | 1:2000 dilution |
| YY1 | ICC/WB | Abcam | ab245364 | 1:100/1:2000 dilution |
| anti-rabbit IgG | WB | bioss | bs-0295G-HRP | 1:5000 dilution |
| anti-mouse IgG | WB | bioss | bs-0296G-HRP | 1:5000 dilution |
| Goat Anti-Rabbit IgG (Alexa Fluor® 555) | IHC-P | Abcam | AB150078 | 1:400 dilution |
| Goat Anti-Mouse IgG (Alexa Fluor® 555) | IHC-P | Abcam | AB150113 | 1:400 dilution |
| Goat Anti-Rabbit IgG (Alexa Fluor® 488) | IHC-P | Abcam | AB150077 | 1:400 dilution |
| Goat Anti-Mouse IgG (Alexa Fluor® 488) | IHC-P | Abcam | AB150114 | 1:400 dilution |

**Table S4. List of the primer sequences that were used**

| Target gene | Forward Primer (5′-3′) | Reverse Primer (5′-3′) |
| --- | --- | --- |
| Pcsk1 | TCTGGTTGTCTGGACCTCTGAGT | CATCAAGCCTGCCCCATTCTTT |
| Gcg | ACTCACAGGGCACATTCACC | TGGCAATGTTGTTCCGGTTC |
| Gip | GAGTTCCGATCCCATGCTAA | TTGTTGTCGGATCTTGTCCA |
| Dpp4 | TTGTGGATAGCAAGCGAGTTG | CACAGCTATTCCGCACTTGAA |
| Mstn | AGTGGATCTAAATGAGGGCAGT | GTTTCCAGGCGCAGCTTAC |
| Slc5a1 | GCCATTGCATTCCTCCCTCT | GCGAGTCCCCAACCCTATTC |
| Slc2a2 | TCATGTCGGTGGGACTTGTG | ACACGTAAGGCCCAAGGAAG |
| Yy1 | ACCTGGCATTGACCTCTCAGAC | CTCATAGCAGAGTTATCCCTGAAC |
| Zdhhc1 | TTGCTAAGGCGGAAGGACTC | TGTTCGCTGACGTGAAAACA |
| Zdhhc2 | CTCTTATGCAGAGAAAGAATTGCTG | GGCAAATGGTGCCTTCCAAT |
| Zdhhc3 | TGTCATGCTGGTTCCATCCC | TTTCCTTTGGGCATCACGGG |
| Zdhhc5 | CCAGGAAGCCCAAGATTGAAGA | GCAGTCCTCGTCTCTTTTTCCG |
| Zdhhc7 | GAGTTCTTGGAAGCCCGGT | GAGGATGGTGCTCGATGTCC |
| Zdhhc8 | CTGTCCAGCTCCATGAGTCG | GCAGATGAGTGGTGGTCAGTT |
| Zdhhc9 | ACGTGAGGAGCGTTCCATTC | TGGTAGCGACTTCTCCCTGA |
| Zdhhc11 | GGCATCAACAAGAACTGGGTC | TAGACAGAGGGCGTGGTAGG |
| Zdhhc13 | TCCCACGTTCCAGGATTTGG | ACAAGTTCCAGCCTGTGTT |
| Zdhhc14 | CTCGCCTCCTGTGTGACAAT | AAGTAAGTAACTACATGCAAGCTG |
| Zdhhc15 | ACAGCAGCCAAACCAGAAGT | GCATCTGCTTCTGGACCTCA |
| Zdhhc17 | GAAACGCTTTCTCCCAGCAT | TCCGACCAAGAGGTTCTCCA |
| Zdhhc18 | CTGATTGACCGGCGAGGATT | CAGAGGGCACAGCTATGGGA |
| Zdhhc20 | GCTTTGCCGCAGGAAATCTAC | GTACGTGGAACTTTGCGCTG |
| Zdhhc21 | TGAAGGGAGGCCCATACGA | GACAATCAAGCCCATGCAGC |
| Zdhhc24 | CGCGGATGCCACTGGTACT | CACGTTGCCCAGCAGATTGAG |
| β-actin | GTACCACCATGTACCCAGGC | AACGCAGCTCAGTAACAGTC |
